# CRISPR-Cas12a REC2–Nuc interactions drive target-strand cleavage and constrain trans cleavage

**DOI:** 10.1093/nar/gkaf988

**Published:** 2025-10-08

**Authors:** Anthony Newman, Aakash Saha, Lora Starrs, Pablo R Arantes, Giulia Palermo, Gaetan Burgio

**Affiliations:** The Shine-Dalgarno Centre for RNA Innovation, Division of Genome Sciences and Cancer, The John Curtin School of Medical Research, The Australian National University, Canberra, ACT 2601, Australia; Department of Bioengineering, University of California, Riverside, 900 University Avenue, Riverside, CA 92512, United States; The Shine-Dalgarno Centre for RNA Innovation, Division of Genome Sciences and Cancer, The John Curtin School of Medical Research, The Australian National University, Canberra, ACT 2601, Australia; Department of Bioengineering, University of California, Riverside, 900 University Avenue, Riverside, CA 92512, United States; Department of Bioengineering, University of California, Riverside, 900 University Avenue, Riverside, CA 92512, United States; Department of Chemistry, University of California, Riverside, 900 University Avenue, Riverside, CA 92512, United States; The Shine-Dalgarno Centre for RNA Innovation, Division of Genome Sciences and Cancer, The John Curtin School of Medical Research, The Australian National University, Canberra, ACT 2601, Australia

## Abstract

CRISPR-Cas12a mediates RNA-guided cleavage of double-stranded DNA in *cis*, after which it remains catalytically active and non-specifically cleaves single-stranded DNA in *trans*. Native host defence by Cas12a employs *cis* cleavage, which can be repurposed for the genome editing of other organisms, and *trans* cleavage can be used for *in vitro* DNA detection. Cas12a orthologues have high structural similarity and a conserved mechanism of DNA cleavage, yet highly different efficacies when applied for genome editing or DNA detection. By comparing three well-characterized Cas12a orthologues (FnCas12a, LbCas12a, and AsCas12a), we sought to determine what drives their different *cis* and *trans* cleavage and how this relates to their applied function. We integrated *in vitro* DNA cleavage kinetics with molecular dynamics simulations, plasmid interference in *Escherichia coli*, and genome editing in human cell lines. We report large differences in *cis* cleavage kinetics between orthologues, which may be driven by dynamic REC2-Nuc interactions. We generated and tested REC2 and Nuc mutants, including a hitherto unstudied ‘Nuc-loop’, integrity of which is critical for the function of Cas12. In total, our *in vitro, in vivo*, and *in silico* survey of Cas12a orthologues highlights key properties that drive their function in biotechnology applications.

## Graphical Abstract

**Graphical Abstract:**
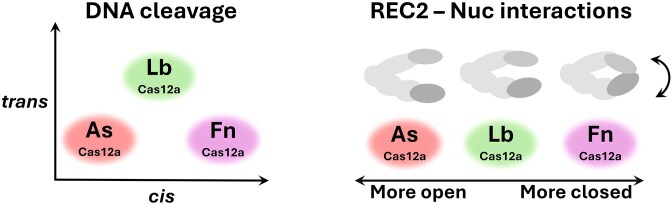

## Introduction

CRISPR-Cas (clustered regularly interspaced short palindromic repeats-CRISPR-associated) are adaptive immune systems in bacteria and archaea that interfere with foreign nucleic acid sequences in an RNA-guided fashion [1]. Cas12a, formerly named Cpf1, is the signature effector of type V-A CRISPR systems [2]. In host defence, Cas12a binds to a guide RNA derived from its CRISPR array—the crRNA (CRISPR RNA)—to effect RNA-programmable cleavage of double-stranded DNA (dsDNA) in *cis* [3–5]. This RNA-guided nuclease activity has been widely employed for the genome editing of eukaryotic cells [5–7]. *In vitro*, Cas12a remains catalytically active after *cis* cleavage, and can cut single-stranded DNA (ssDNA), RNA, and nick dsDNA [8–10]. The target-activated *trans* cleavage of Cas12a underlies its applications for molecular detection. With reverse transcription and aptamer strategies, RNA, proteins, small molecules, and even heavy metals can also be detected using Cas12a [11, 12].

**Figure 1.**
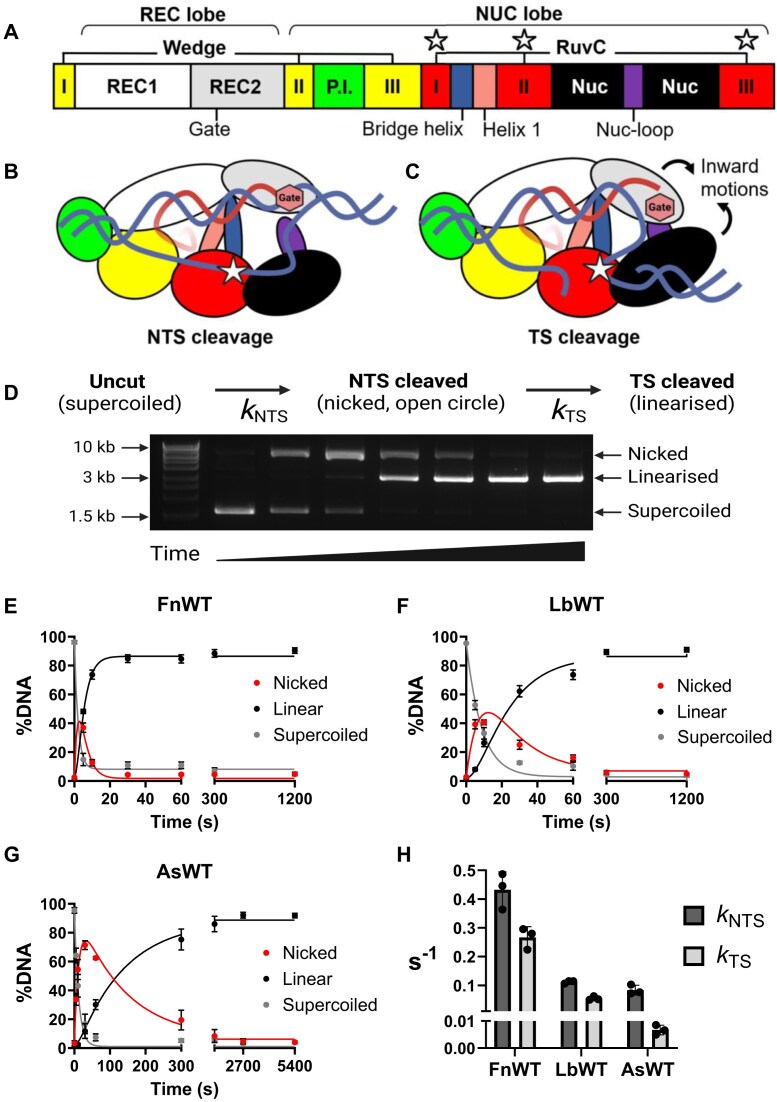
(**A**) General domain organization of Cas12a orthologues (not to scale). Key residues and motifs are highlighted: the REC2 ‘gate’, the bridge helix and helix 1—which comprise the ‘BH’ domain, the Nuc-loop (purple), and the RuvC active site residues (white stars). (B, C) Model of Cas12a tertiary structure bound to crRNA (red) and dsDNA (blue). Depicted is the *cis* cleavage mechanism, where the non-target strand (NTS) is cleaved first (**B**), followed by TS cleavage (**C**). Key residues and motifs in this mechanism are highlighted: REC2 ‘gate’ (red hexagon), Nuc-loop (purple), and RuvC active site (white star). (**D**) Schematic showing changes in plasmid DNA topology with sequential NTS and TS cleavage. Example agarose gel of plasmid cleavage over time (image over-exposed to show faint DNA bands). DNA cleavage by Cas12a results in evident changes in plasmid DNA topology, from the uncut and negatively supercoiled form (migrates at ∼1.5 kb), to the nicked open-circle form (migrates at ∼9 kb), to the linearized form (migrates at ∼3 kb). Quantification of DNA fractions (nicked, linearized, supercoiled) over time, when incubated with (**E**) WT FnCas12a, (**F**) WT LbCas12a, and (**G**) WT AsCas12a—note longer time points. Dots show mean %DNA, error bars ± s.d. The solid line shows the fit of the kinetic model, using kinetic values averaged from the three replicates. (**H**) *Cis* cleavage kinetics for WT FnCas12a, LbCas12a, and AsCas12a. Bar shows mean rate constant of NTS (dark grey) and TS cleavage (light grey), and error bars show ± s.d. Dots show individual replicates.

Cas12a assumes a ‘crab-claw’ structure of two lobes, with nuclease (NUC) and recognition (REC) lobes that effect their eponymous functions [4, 13–18] (Fig. 1A). Cas12a scans dsDNA and initiates R-loop formation at protospacer-adjacent motifs (PAMs) [19]. The REC lobe recognizes a matching DNA target site by stable R-loop formation between crRNA and a hybridized DNA strand (target strand, TS) [14, 18, 20]. Docking of the flexible REC2 domain to the bridge helix (BH) domain activates the distant RuvC domain in the NUC lobe by stabilizing the open conformation of a RuvC-occluding ‘lid’ loop [18, 20–24]. This narrow active site cleft can only sterically accommodate ssDNA, so dsDNA cleavage by Cas12a has to occur via sequential cleavage of unwound strands [4, 13, 25].

The non-hybridized DNA strand (NTS) is coordinated close to the RuvC and in the required 5′ to 3′ polarity for in-line nucleophilic attack by the RuvC, while the TS is hybridized to the crRNA, stuck to the REC lobe far from the active site and in the opposite polarity [4, 13, 15, 16] (Fig. 1B and C). Consequently, kinetic studies of sequential DNA strand cleavage have determined NTS cleavage is 2–20× faster than TS cleavage [14, 18, 20, 23, 26, 27]. The NTS occludes the TS from the active site until it is cleaved, making an obligatory sequential cleavage mechanism of NTS cleavage preceding TS [13, 25] (Fig. 1B and C).

Structures of Cas12a show the scissile phosphate of the TS is some 25 Å from the RuvC active site [13–16]. Dynamic ‘pinching’ motions of the REC2 and Nuc domains have been observed in single-molecule FRET studies and molecular dynamics (MD) simulations [14, 24, 28–31], motions which shorten the distance required for the TS to traverse. A key aromatic ‘gate’ residue in the REC2 stacks at the 20th position of the crRNA–TS heteroduplex, regulating the length of the R-loop and constraining the flexible fraying of the 3′ R-loop junction [25, 26] (Fig. 1B and C).

After *cis* cleavage, Cas12a remains stably bound to the PAM-proximal fragment of dsDNA containing the entire 20 bp crRNA:TS heteroduplex used for target recognition [19]. This ternary complex remains catalytically active to cleave nucleic acids in *trans* [9, 10].

This mechanism of *cis* and *trans* cleavage is considered to be consistent across Cas12a orthologues. Yet, when it comes to applying these enzymatic capabilities to genome editing and molecular detection, efficacies vary greatly between orthologues. These differences raise the question—what makes an effective Cas12a in which context? In a time when abundant Cas12a genes can be obtained from sampling environmental DNA [32], from genetic databases [33], and even designed *de novo* [34], resolving this question would greatly improve the efficiency of finding or engineering improved Cas12a nucleases [35].

To explore this question, we undertook a comparative study of three well-characterized Cas12a orthologues, from *Acidaminococcus*sp.*BV3L6* (AsCas12a), *Lachnospiraceae bacterium ND2006* (LbCas12a), and *Francisella tularensis*subsp.*novicida U112* (FnCas12a). When compared side-by-side in human cell lines, editing by AsCas12a and LbCas12a is more robust than FnCas12a, across a range of PAMs and target sites [5, 6]. For *trans* cleavage activity, it is LbCas12a that has more robust activity than AsCas12a and FnCas12a [9, 10, 33].

AsCas12a, LbCas12a, and FnCas12a have high structural similarity (<3 Å RMSD) with <50% sequence similarity [4, 13–18]. Given their shared mechanism of target DNA cleavage, they each ‘solve’ the same molecular problem with somewhat different amino acid sequences. We suspected this divergence causes their different performance in applied settings.

To interrogate this hypothesis, we generated mutations in the REC2 and Nuc domains to explore what drives their DNA cleavage kinetics. We identified an uncharacterized ‘Nuc-loop’ as a structural element that traverses the distance between Nuc and REC2 domains. This loop is present across Cas12a orthologues but varies considerably in length and amino acid sequence. Furthermore, the Nuc-loop is unresolved in most experimentally determined structures of Cas12a, suggesting it may be highly dynamic. Only recently have cryo-EM structures captured the Nuc-loop in the process of coordinating DNA strands for cleavage [18].

Across FnCas12a, LbCas12a, and AsCas12a, we thoroughly characterize REC2 and Nuc-loop mutants for their *cis* and *trans* cleavage kinetics, and their ability to interfere with plasmid transformation in *Escherichia coli* and edit genes in human cell lines. We find apparent trade-offs between NTS, TS, and *trans* cleavage, which are driven by REC2 ‘gate’ and Nuc-loop interactions. Although mutagenesis could modulate *cis* cleavage rates five-fold, there remained very large differences in between Cas12a orthologues, with FnCas12a displaying extremely rapid and robust DNA cleavage. To resolve this conundrum, and elucidate the dynamic role of the Nuc-loop, we conducted MD simulations to compare the properties of REC2–Nuc dynamics between Cas12a orthologues. Together with recent cryo-EM and MD results, this simulation shows the Nuc-loop makes dynamic interactions with the REC2 and the crRNA–TS heteroduplex. Furthermore, we found large differences in REC2–Nuc distance distributions, which may underwrite their different efficiencies of allosterically activating DNA catalysis. In total, this work advances our understanding of the mechanisms of nuclease activities of Cas12a orthologues.

## Materials and methods

### Cloning

The coding sequences of WT FnCas12a and AsCas12a were cloned from parent vectors (Addgene #90094 and #90095, respectively) into a pET21_6His_2NLS vector that was a kind gift of Wolfe Lab (Addgene #114366), as described previously [21]. Similarly, plasmid interference ‘locus’ plasmid was cloned with the In-Fusion kit (Takara Bio), as previously described [21].

Mutant sequences, such as Cas12a mutants, were generated using the Q5 site-directed mutagenesis kit according to the manufacturer’s instructions (New England Biolabs—NEB), and sequences verified with Sanger sequencing (Biomolecular Resource Facility, ANU) (Supplementary Table S5—primers, Supplementary Table S6—‘locus’ oligonucleotides).

### Protein purification

All WT and mutant Cas12a proteins were purified with the following protocol. Plasmids were transformed into T7-express chemically competent cells (NEB), colonies picked, and small volumes (∼5 ml) grown overnight in Luria Broth supplemented with 100 μg/ml ampicillin. Overnight cultures were used to inoculate a 250 ml culture, grown at 37°C for ∼2 h in baffled flasks and vigorous shaking, until OD_600_ ∼0.6. Flasks were put on ice for 30–45 min before addition of 1 mM isopropylthio-β-galactoside (IPTG), then transferred to an 18°C incubator for shaking at 200 rpm overnight.

Expression cultures were centrifuged for 10 min at 5000 × *g*, and pellets resuspended in Lysis Buffer (50 mM Tris–HCl, pH 7.5, 500 mM NaCl, 5% glycerol, 1 mM dithiothreitol [DTT]), with addition of one ‘cOmplete protease inhibitor tablet’ (Roche) per 50 ml of resuspension. Cells were lysed with sonication (Branson Sonifier), and supernatant clarified with 2 × 30 min centrifuge spins at 13 500 × *g*. We performed metal affinity chromatography by loading the supernatant on an equilibrated Ni-NTA HisTrap column (GE Healthcare, 5 ml) with an AKTA Explorer (GE Healthcare), washed with Buffer A (50 mM Tris–HCl, pH 7.5, 500 mM NaCl, 20 mM imidazole, 5% glycerol), and eluted with a stepwise addition of Buffer B (50 mM Tris–HCl, pH 7.5, 500 mM NaCl, 500 mM imidazole, 5% glycerol). Fractions were analysed with Sodium dodecyl sulphate–polyacrylamide gel electrophoresis (SDS–PAGE), and peak fractions containing Cas12a were pooled, and diluted 2.5× with diluting buffer (50 mM Tris–HCl, pH 7.5, 5% glycerol) to achieve 200 mM NaCl for cation exchange chromatography. After loading on a HiTrap Heparin column (GE Healthcare, 5 ml) pre-equilibrated with Buffer H-A (50 mM Tris–HCl, pH 7.5, 200 mM NaCl, 5% glycerol), elution was performed with a linear gradient of Buffer H-B (50 mM Tris–HCl, pH 7.5, 1 M NaCl, 5% glycerol). Again, fractions containing Cas12a were determined by SDS–PAGE and concentrated to a small volume (∼500 μl) by centrifugal molecular-weight cut-off tubes (30 kDA, Pierce Thermo Fisher). Concentrated protein was then buffer-exchanged into storage buffer (50 mM Tris–HCl, pH 7.5, 500 mM NaCl, 50% glycerol, 1 mM DTT) with 0.5 ml centrifugal molecular-weight cutoff tubes (Millipore). Protein concentration was estimated with a Nanodrop spectrophotometer (Thermo Fisher) using extinction coefficients calculated with Expasy ProtParam [36] and stored at −20°C. Yields of Cas12a varied from 2–8 g/l of expression culture.

### Protein thermostability assay

Melt curves were conducted in 40× SYPRO Orange dye (Thermo Fisher) and a StepOnePlus quantitative-PCR machine (Applied Biosystems), following the manufacturer’s detailed protocol (Thermo Fisher). Cas12a proteins (in storage buffer) were diluted in nuclease-free water (Ambion) to a concentration of ∼1 μg per well, 3 × 20 μl replicates were added to a MicroAmp Fast 96-well Reaction Plate (Applied Biosystems), and fluorescence monitored at 1°C increments from 25 to 99°C. Melting temperature was defined as the fluorescence peak.

### Cis cleavage kinetics

In this assay, Cas12a was complexed with a crRNA targeting a site in a negatively supercoiled plasmid. RNAs were ordered from Integrated DNA Technologies (IDT) and resuspended in IDTE buffer (IDT) (Supplementary Table S7—crRNA sequences). Cas12a–crRNA complexes were assembled by incubation at 25°C for 10 min. Complexes were diluted in 1× cleavage buffer (10 mM Tris–HCl, pH 7.5, 10 mM MgCl2, 50 mM NaCl, 5 μg/ml bovine serum albumin [BSA], 0.1 mM DTT) to a final concentration of 100 nM, and equilibrated at 30°C on a thermocycler block prior to addition of target DNA.

‘DNA solution’ containing target plasmid DNA was diluted in 1× cleavage buffer to a final concentration of 10 nM, and also equilibrated at 30°C. Equal volumes (5 μl) of Cas12a complex and DNA solution were mixed and incubated for the set time-points, and reaction quenched with addition of 5 μl ‘STOP solution’ [15% v/v proteinase K (Bioline), 250 mM ethylenediaminetetraacetic acid, 50% v/v 5× loading dye (Bioline), in nuclease-free water (Ambion)]. To remove DNA-bound Cas12a ribonucleoprotein, all samples were incubated at 55°C for 30 min after addition of STOP solution.

DNA products were separated by gel electrophoresis (100 V for 40 min) on a 1.5% agarose gel pre-stained with 0.5× GelRed (Biotium). Gels were imaged with a Quantum geldoc (Vilber) with short exposure times to avoid oversaturation, and DNA band intensity quantified with ImageJ [37] (NIH). Changes in target plasmid topology were validated by nuclease digestion by Nt.BspQI and EcoRI (Supplementary Fig. S1), using the manufacturer’s protocol (NEB). Efficiency of GelRed binding to different DNA topologies was determined as per [38], by a dilution series of equal parts supercoiled, nicked, and linear DNA. Nicked and linearized plasmid DNA was generated by Nt.BspQI and EcoRI digestion of highly pure pUC19 (NEB). The supercoiled sample was generated by incubation without nuclease. Three replicates were performed, and pixel intensity quantified with ImageJ [37] (NIH). This determined less efficient binding to supercoiled DNA (Supplementary Figs. S2 and S3). Correction factors of 0.797, 0.855, and 1.74 were determined to normalize signal from the nicked, linear, and supercoiled bands, respectively (Supplementary Fig. S3). Corrected pixel numbers from DNA fractions ‘nicked’, ‘linear’, and ‘supercoiled’ were summed, from which was calculated the percentage of nicked/linear/supercoiled DNA fractions. Percentage of DNA fraction at time points was used as input for kinetic modelling, where each replicate was individually fitted. Three replicates were performed for each Cas12a nuclease.

### Modelling rates of sequential DNA strand cleavage

The rate of change between DNA fractions was then modelled to obtain *k*_obs_ for both NTS and TS cleavage, where ka = *k*_NTS_ and kb = *k*_TS_. Rates were fitted in Berkeley Madonna [39], using the following equations, as previously detailed in the literature [26, 27, 40]. This models the sequential conversion of SC to NICK to LIN, while allowing incomplete reaction products unconverted-NICK (ucNICK) and unconverted-SC (ucSC) to accumulate.

d/dt (SC) = −ka*SC − kini*SC

d/dt (ucSC) = kini*SC

d/dt (NICK) = ka*SC − kb*NICK − kini2*NICK −ka*NICK1

d/dt (NICK1) = −ka*NICK1 − kini*NICK1

d/dt (ucNICK) = kini2*NICK + kini*NICK1

d/dt (LIN) = kb*NICK # used to plot %linear

TotSC = SC + ucSC # used to plot %supercoiled

TotNICK = NICK + NICK1 + ucNICK # used to plot %nicked

Init SC = input initial %SC from dataset

init LIN = input initial %LIN from dataset

init NICK1 = input initial %NICK from dataset

init NICK = 0

init ucSC = 0

init ucNICK = 0

ka = 0.50*

kb = 0.50*

kini = 0.01* # inactivation rate of NICK1 to ucNICK, SC to ucSC

kini2 = 0.01* # inactivation rate of NICK into ucNICK

Where variables ‘SC’, ‘NICK’, and ‘LIN’ were fitted to their corresponding dataset, being the percentage of nicked/linear/supercoiled DNA at timepoints. Variables marked with an asterisk (*) were fitted by the software.

### Trans cleavage assays

Cas12a–crRNA complexes were prepared as described for *cis* cleavage (10 nM final concentration), with either 20 or 23 nt spacer-length crRNAs (Supplementary Table S7). *Cis* cleavage was performed by addition of 1 nM full-length or truncated target-strand DNA (Supplementary Table S6) in 1× cleavage buffer, followed by incubation at 30°C for 45 min. This active complex was then further diluted in 1× cleavage buffer, and 50 μl added to wells of a flat-clear-bottom black fluorescence 96-well plate (Thermo Fisher). Fluorescent-quencher reporter ssDNA (Supplementary Table S6) was prepared in 1× cleavage buffer to a final concentration of 75 nM, 50 μl of which was added into each well, using the dispenser pump of a Victor Nivo plate reader (PerkinElmer). Excitation and emission filters of 480/30 and 530/30 nm, respectively, were used to measure fluorescence over time. Three replicates were performed per condition.

### Structural analysis

Protein Data Bank (PDB) files for the accession codes indicated were obtained from rcsb.org, and structural alignment performed in VMD [41], using the STAMP algorithm [42]. Predicted Cas12a structures were obtained from Alphafold/EMBL [43, 44], to fill structural elements not modelled in experimental structures of LbCas12a. UniProt identifiers were A0Q7Q2 for FnCas12a, U2UMQ6 for AsCas12a, and A0A182DWE3 for LbCas12a.

### Plasmid interference assay

This assay used three plasmids, one expressing Cas12a under a T7 promoter (AmpR), another encoding a crRNA under a T7 promoter (CmR) with a spacer sequence matching a third ‘target’ plasmid (KanR).

To achieve high rates of plasmid transformation, chemically competent T7Express cells (NEB) were made harbouring both the Target plasmid, and either the crRNA expressing (+ crRNA) or empty vector (no crRNA). These ± crRNA strains were transformed with 20 ng of Cas12a plasmid by heat shock at 42°C and recovered with SOC media at 37°C for 30 min. Serial dilutions were plated onto triple selective media (100 μg/ml ampicillin, 50 μg/ml kanamycin, 25 μg/ml chloramphenicol) containing 0.5 mM IPTG. Plates were incubated overnight at 37°C for ∼16 h, and colonies counted. Three replicates were performed for each transformation. Statistical significance was calculated by two-way ANOVA followed by Tukey’s multiple comparisons test, using GraphPad Prism 10 (GraphPad Software, www.graphpad.com).

### Human cell line editing

Human genome targets with previously identified off-target sites were chosen for gene editing experiments [6, 7, 45]. The target sites respectively have the canonical PAM motifs, TTTA, TTTC, and TTTG, to minimize PAM bias in editing efficiencies between Cas12a orthologues [6, 16, 46–48]. The most-represented off-target sites identified by GUIDE-seq and DIGENOME-seq were chosen for high-throughput sequencing [6, 7, 45]. crRNAs were ordered as HPLC-purified RNA (IDT) (Supplementary Table S7).

HEK293T, A549, and Jurkat cell lines were obtained from the American Type Culture Collection and tested free of mycoplasma infection. HEK293T were cultured in high glucose Dulbecco’s Modified Eagle Medium (Gibco) supplemented with 10% Fetal Bovine Serum (FBS) and 1× Penicillin-Streptomycin-Glutamine (Gibco). Jurkat cells were cultured in RPMI-1640 supplemented with 10% FBS and 1× Penicillin-Streptomycin-Glutamine (Gibco). A549 were cultured in Ham’s F-12K (Kaighn’s) Medium (Gibco) supplemented with 10% FBS and 1× Penicillin-Streptomycin (Gibco). Cells were maintained at 37°C with 5% CO_2_ in a humidified atmosphere and transfected at passage 10.

Cas12a proteins were assembled with their cognate crRNA, targeting either DNMT1–3, DNMT1–7, or AGBL1. Each RNP reaction consisted of 0.575 μM crRNA with 32 pM of Cas12a. The crRNA and Cas12a were complexed in 2.2 μl Neon Transfection System ‘R’ resuspension buffer (Invitrogen) at 37°C for 5 min and left at room temperature post-complexing.

Electroporation was conducted using Neon Transfection System (Invitrogen) according to the manufacturer’s protocol, with the following modification that all three cell lines were resuspended in Neon Transfection System ‘R’ resuspension buffer (Invitrogen) to a concentration of 1 × 10^7^/ml.

For each electroporation reaction, 1 × 10^5^ cells prepared earlier were incubated with 1× RNP at 37°C for 5 min before being electroporated using the 10 μl Invitrogen Neon Transfection System. Electroporation protocols for cell lines were HEK293T; 1150 volts, 20 ms, 2 pulses; Jurkat; 1325 volts, 10 ms, 3 pulses; A549; 1230 volts, 30 ms, 2 pulses. Two reactions were seeded per well, in a 24-well plate. Cells were recovered in complete medium at 37°C with 5% CO_2_ for 72 h. Controls for each cell line included no electroporation, and electroporation without RNP—labelled ‘No Cas12a’. Two replicates were performed for each control and three for each Cas12a.

Samples were harvested at 72 h post transfection, including growth media to capture all cells, dead or alive. Cells were pelleted at 500 × *g* for 2 min at room temperature, then washed with 1× PBS. Cells were again centrifuged at 500 × *g* for 2 min at room temperature, PBS was removed, and samples were then stored at −20°C prior to DNA extraction. Samples were thawed on ice and genomic DNA was extracted using the ISOLATE II Genomic DNA Kit (Meridian Bioscience), and following the manufacturer’s instructions, with the sole modification of eluting twice in with the same 100 μl of elution buffer.

### Quantification of genome editing by high-throughput sequencing

Primers were designed for high-throughput sequencing of identified on and off-target sites of DNMT1–3, DNMT1–7, and AGBL1; positioned ∼125 bp upstream and downstream of the target site, resulting in ∼250 bp amplicon (primers in Supplementary Table S8). Primers and target DNA were dispensed in 384 well plates, and Illumina Ampliseq used to perform a paired-end 250 bp library preparation.

Quality control was performed on the ∼32 million reads obtained, using FastQC [49]. The reads were then analysed by CRISPResso2 [50], using the hg38 human genome as reference using the following parameters: –cleavage_offset 1 –quantification_window_size 20 –ignore_substitutions –default_min_aln_score 50. Samples with <1000 mapped reads were discarded. Insertions and deletions in this window were combined to calculated total percentage of indels at a given on or off-target site. Statistical significance was calculated by two-way ANOVA followed by Tukey’s multiple comparisons test, using GraphPad Prism 10 (GraphPad Software).

### Structural models for simulation

Molecular simulations were based on three structures of Cas12a across different species: (i) the cryo-EM structure of FnCas12a (PDB: 6GTG) [14] at 3.27 Å resolution, (ii) the X-ray structure of AsCas12a from (PDB: 5B43) [15] at 2.80 Å, (iii) the X-ray structure of LbCas12a (PDB: 5XUS) [16] at 2.5 Å. Missing residues were added by homology modelling using SwissModel [51]. All systems were embedded in explicit waters, and counterions were added to neutralize the total charge, leading to periodic cells of ∼138 × 149 × 167 Å^3^ and ∼307 000 atoms for each system.

### Molecular dynamics simulations

MD simulations were performed using a protocol tailored for RNA/DNA nucleases using the Amber ff19SB force field [52], including the ff99bsc1 corrections for DNA [53] and ff99bsc0+χOL3 corrections for RNA [54, 55]. The TIP3P model was employed for explicit water molecules [56], and the Li & Merz 12–6 model of non-bonded interactions was used for Mg^2+^ ions [57]. We have extensively employed these force field models in computational studies of CRISPR-Cas systems [29], showing also that they perform well for long timescale simulations [24]. The Li & Merz model also reported a good description of Mg^2+^ bound sites, in agreement with quantum/classical simulations [58]. An integration time step of 2 fs was employed. All bond lengths involving hydrogen atoms were constrained using the SHAKE algorithm [59]. Temperature control (300 K) was performed via Langevin dynamics [60], with a collision frequency *γ* = 1. Pressure control was accomplished by coupling the system to a Berendsen barostat [61], at a reference pressure of 1 atm and with a relaxation time of 2 ps.

The systems were subjected to energy minimization to relax water molecules and counterions, keeping the protein, the RNA, DNA, and Mg^2+^ ions fixed with harmonic position restraints of 300 kcal/mol·Å^2^. Then, the systems were heated up from 0 to 100 K in a canonical ensemble (NVT), 120 by running two simulations of 5 ps each, imposing position restraints of 100 kcal/mol·Å^2^ on the above-mentioned elements of the system. The temperature was further increased up to 200 K in ∼100 ps of MD in the isothermal-isobaric ensemble (NPT), reducing the restraint to 25 kcal/mol·Å^2^. Subsequently, all restraints were released, and the temperature of the systems was raised up to 300 K in a single NPT simulation of 500 ps. After ∼ 1.1 ns of equilibration, ∼10 ns of NPT runs were carried out allowing the density of the systems to stabilize around 1.01 g cm^−3^. Finally, production runs were carried out in the NVT ensemble in four replicates, collecting ∼1 μs for each replicate. These simulations were performed using the GPU-empowered version of AMBER 20.

For distance and contact analyses, domains were defined as follows: FnCas12a, REC2 (340–591), and Nuc (1079–1254); LbCas12a, REC2 (283–521), and Nuc (998–1179); and AsCas12a, REC2 (321–526), and Nuc (1067–1262). Distance analysis considered the centre of mass of the selected regions, and a contact was considered when the distance between two heavy atoms among the regions of interest was <3.5 Å. Amino acids involved in contacts were identified by visual inspection of trajectories. Kernel density estimation plots were employed to visualize the probability density of REC2-Nuc distances and contacts, using the ‘kdeplot’ function from the seaborn library, a statistical data visualization package in Python [62].

## Results

### Cas12a orthologues display distinctly different rates of sequential strand cleavage

Central to the natural or applied functions of Cas12a is *cis* cleavage. The kinetics of sequential DNA strand cleavage have been determined for wild-type FnCas12a, LbCas12a, and AsCas12a, and have yielded rate constants that vary by several orders of magnitude (Supplementary Table S1) [14, 18, 20, 23, 26–28]. This can be attributed to a number of factors known to affect *cis* cleavage kinetics; temperature [63], DNA substrate topology [27], and magnesium ion concentration [20, 28]. Thus, we wished to undertake kinetic comparisons in conditions at which each three orthologues had previously been determined to be maximally active [64]; 30°C, pH 7.5, 50 mM NaCl (plus 10 mM Tris–HCl, 10 mM MgCl_2_, 5 μg/ml BSA, 0.1 mM DTT).

We determined the *cis* cleavage kinetics of the three Cas12a orthologues using a plasmid cleavage assay [26, 27, 40]. Briefly, the sequential DNA strand cleavage of a negatively supercoiled plasmid causes sequential changes in plasmid DNA topology, transitions that are visible in gel electrophoresis [65] (Fig. 1B–D and Supplementary Fig. S1). NTS cleavage relaxes the supercoiled plasmid into the open-circle form, and TS cleavage converts the open-circle to the linearized form [26, 27, 40]. Quantification of these topological changes over time allows fitting of strand cleavage rates [26, 27, 40].

In this time-course assay of plasmid DNA cleavage, FnCas12a exhibited the fastest DNA cleavage, with the fraction of linearized DNA plateauing at 30 s (Fig. 1E). LbCas12a linearized the target plasmid by 300 s (Fig. 1F), and AsCas12a by the 2700 s time-point (45 min, Fig. 1G). Fitting a sequential-strand cleavage model to this time-course assay data yielded rate constants for NTS and TS cleavage [26, 27, 40] (Fig. 1H).

This showed the large time differences in linearizing plasmid DNA are driven by very different *cis* cleavage kinetics (Fig. 1G). FnCas12a exhibited a *k*_NTS_ 3.9x faster than LbCas12a, which in turn had a *k*_NTS_ 1.3× faster than AsCas12a (Fig. 1G and Supplementary Table S1). The differences were greater with *k*_TS_; FnCas12a was 4.9× faster than LbCas12a, which was 7.9× faster than AsCas12a (Fig. 1G and Supplementary Table S1).

This order of *cis* cleavage speed, where FnCas12a > LbCas12a > AsCas12a, is evident in other reports (comparing FnCas12a, LbCas12a, and AsCas12a [19]; and between FnCas12a and AsCas12a [25]. Of these three orthologues, FnCas12a is generally reported to have lower gene editing efficiency in human cell lines, and weaker *trans* cleavage [5, 6, 9, 10, 33]. We therefore expected FnCas12a to have a defect in *cis* cleavage. But in the conditions tested, FnCas12a has the most robust *cis* cleavage. This raises the question; what drives the different *cis* cleavage kinetics of Cas12a orthologues, and how does it relate to genome editing and DNA detection?

### REC2 domain mutations reduce NTS and trans cleavage rates

The NTS cleavage mechanism of Cas12a is straightforward; a groove of DNA-binding residues across the Wedge, RuvC, and Nuc domains guides the NTS into the RuvC active site in the correct 5′ to 3′ polarity for in-line nucleophilic attack [4, 13, 14, 18]. The mechanism of TS cleavage is less simple, the scissile phosphate must traverse over 20 Å and twist 180° to enter the RuvC with the correct polarity [4, 13, 25, 26]. This conformation of the TS is allowed by unwinding at the 3′ end of the crRNA:TS R-loop [14, 18, 25, 26].

A ‘gate’ residue in the REC2 plays a key role in this process, stacking after the 20th position of the crRNA:TS heteroduplex and regulating the length of the R-loop [4, 13–16, 18, 26]. Removing this stacking interaction by alanine substitution increased TS cleavage rates in LbCas12a [26]. Despite increased *k*_TS_, this mutant displayed slower *trans* cleavage [66, 67].

We were intrigued by this apparent trade-off between TS cleavage and *trans* cleavage. To explore this, we generated alanine substitutions of the REC2 gate for three Cas12a orthologues and assayed their *cis* and *trans* cleavage kinetics.

**Figure 2.**
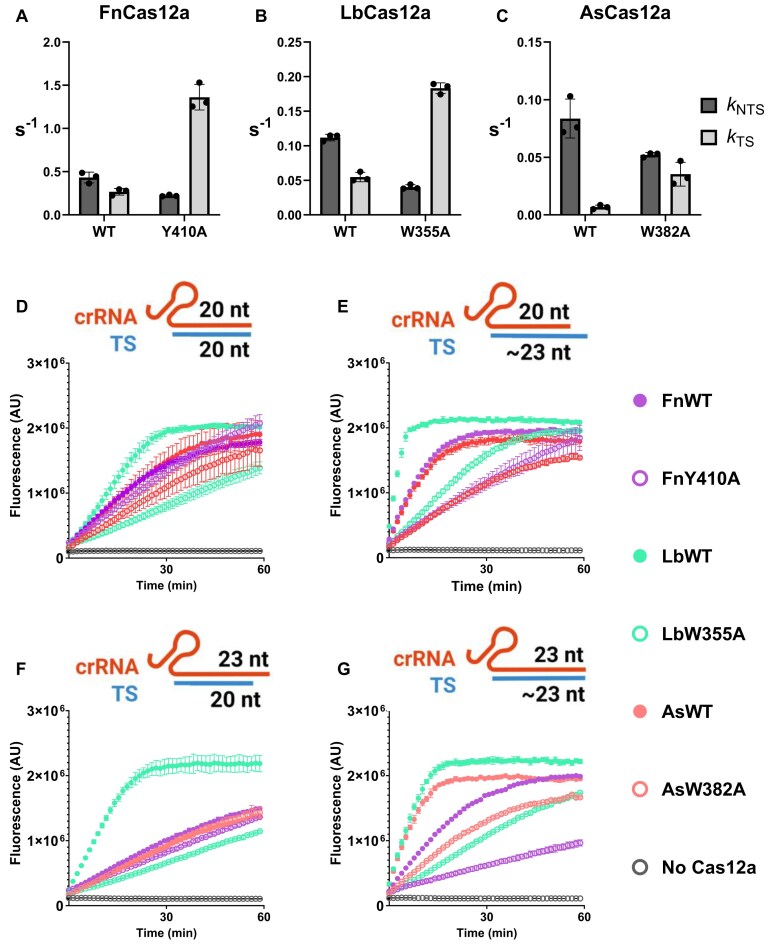
*Cis* cleavage kinetics for WT and REC2 mutants of (**A**) FnCas12a, (**B**) LbCas12a, (**C**) and AsCas12a. Bar shows mean rate constant of NTS (dark grey) and TS cleavage (light grey), and error bars show ± s.d. Dots show individual replicates. (**D**–**G**) *Trans* cleavage curves when activated with the combination of crRNA and TS indicated. Dots show mean ± s.d., for FnCas12a WT (solid purple) and Y410A (hollow purple), LbCas12a WT (solid green) and W355A (hollow green), AsCas12a WT (solid red) and W382A (hollow red), and No Cas12a (hollow grey).

The time-course plasmid cleavage assay was performed for FnY410A, LbW355A, and AsW382A (Supplementary Figs. S4–S5). For FnCas12a, *k*_NTS_ decreased by 2.0x, and *k*_TS_ increased by 5.1× (Fig. 2A). For LbCas12a, *k*_NTS_ decreased by 2.8× while *k*_TS_ increased by 3.3×, consistent with previous reports [26] (Fig. 2B). For AsCas12a, *k*_NTS_ decreased by 1.6×, and *k*_TS_ increased by 5.0× (Fig. 2C). Given *cis* cleavage kinetics vary by orders of magnitude between wild-type orthologues (Supplementary Table S2), this effect is remarkably similar across REC2 mutants. Interestingly, increased *k*_TS_ came at a cost of decreased *k*_NTS_ (Fig. 2A–C and Supplementary Table S2). This suggests that the increased TS-loading of REC2 mutants can interfere with NTS-loading.

Having replicated the faster *k*_TS_ of REC2 ‘gate’ mutants, we aimed to test their *trans* cleavage kinetics. LbW355A has been shown to have reduced *trans* cleavage, suggested to be a result of steric hindrance between loading of the already-cut TS and *trans* ssDNA substrates [66, 67]. To test this, we compared the *trans* cleavage of WT and REC2 mutants when activated by a truncated or full-length TS. When activated by a TS truncated at the 20th position (relative to the PAM), steric hindrance near the active site should be minimal, and WT and REC2 mutants should have similar *trans* cleavage activity. However with a longer TS, the faster TS-loading of REC2 mutants should result in slower *trans* cleavage than WT.

We assembled Cas12a–crRNA complexes with TS ssDNA that was either full-length (96 nt), or truncated at the 20th position relative to the PAM (68 nt) (Supplementary Table S6). These ‘TS-loading/*trans*-active’ complexes were made by a 45-min *cis* cleavage reaction. In this experiment, the full-length TS would be cleaved and trimmed to ∼22–24 nucleotides, depending on the orthologue [20, 22, 25, 26].

We first compared Cas12a ternary complexes with a 20 nt crRNA spacer sequence and truncated TS (Fig. 2D). In this condition, FnY410A and AsW382A had similar rates of *trans* cleavage activity to their WT enzyme (Fig. 2D). However, when complexed with a full-length TS, FnY410A and AsW382A had slower *trans* cleavage activity compared to WT (Fig. 2E). LbW355A showed slower cleavage than WT for both combinations (Fig. 2D and E).

Previous work has also demonstrated that 3′ extension of the crRNA past the 20th position also influences *cis* and *trans* cleavage rates [10, 26, 68]. We repeated the truncated/full-length TS comparison, but with crRNAs consisting of a 23 nt spacer sequence. To compare pseudo-first order *trans* cleavage rates, we fitted a linear regression to the first 300 s of fluorescence curves [35, 67] (Supplementary Fig. S6). We observed a similar pattern as with 20 nt crRNAs; where REC2 mutants of FnCas12a and AsCas12a have similar-to-WT rates of *trans* cleavage with the truncated TS (Fig. 2F), and decreased *trans* cleavage with the full-length TS (Fig. 2G). However, LbWT showed faster *trans* cleavage than LbW355A for all combinations of crRNA and TS, suggesting steric hindrance by the TS is not universal (Fig. 2D–G). Overall, these data suggest that TS-loading can indeed slow *trans* cleavage.

However, REC2 mutants FnY410A and LbW355A showed more incomplete DNA cleavage than WT, with greater amount of uncut plasmid DNA at the endpoint of *cis* cleavage reactions (Supplementary Figs S4 and S5). We tested WT and REC2 mutants for their ability to interfere with plasmids in *E. coli*, and found no defect in their function (Supplementary Fig. S7A and B). A previous study of LbW355A suggested incomplete target DNA cleavage was due lesser stability, as inferred from breakdown products seen in SDS–PAGE [26], however, we found no defect in the thermostability of WT or REC2 mutant *apo* proteins (Supplementary Fig. S7C). However, previous work showed the AsW382A mutant had decreased gene editing activity in human cell lines [15], this suggests that although REC2 mutants can retain activity *in vitro* and in plasmid interference, they nonetheless have a defect relative to WT.

### Nuc-loops are critical to Cas12a function

The REC2 ‘gate’ appears to regulate *k*_TS_, yet there remains a ∼20 Å distance for the TS to traverse from REC2 to RuvC. Structural data at the time showed a lack of electron density between the REC2 and Nuc domains [4, 13–16]—with the exception of a single structure [14]. A ‘transient state’ ternary structure of FnCas12a captured a loop extending from the bulk of the Nuc domain towards the 3′ end of the crRNA–TS heteroduplex [14]. We hypothesized this Nuc-loop could be key in the TS-loading mechanism of Cas12a orthologues.

**Figure 3.**
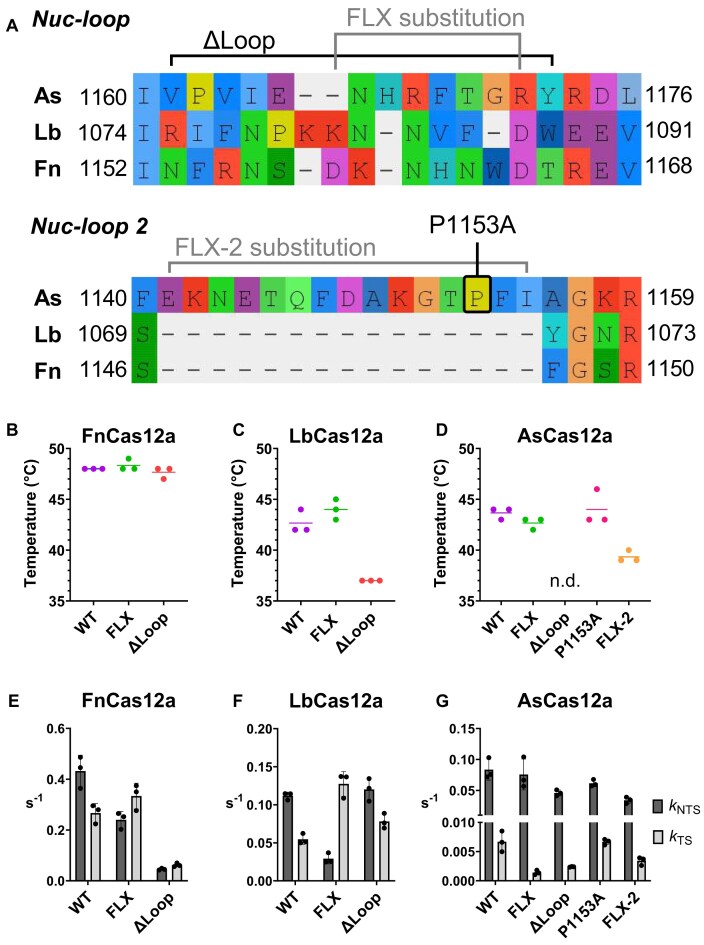
(**A**) Amino acid sequence of AsCas12a (8SFQ), LbCas12a (AF2), and FnCas12a (6GTG), from STAMP structural alignment. Regions mutated in the Nuc-loop are annotated; where ‘FLX’ indicates regions substituted for glycine-serine repeats (grey bracket), ‘ΔLoop’ indicates regions deleted (black bracket). Regions mutated in Nuc-loop 2 are annotated; alanine substitution of P1153 (black box) and FLX-2 truncation/substitution to glycine-serine-glycine (grey bracket). Precise mutations are enumerated in Supplementary Table S3. (**B**–**D**) Thermostability assay, dots show melting temperature of individual replicates, line shows mean. *Cis* cleavage kinetics for WT and Nuc-loop mutants of (**E**) FnCas12a, (**F**) LbCas12a, (**G**) and AsCas12a. Bar shows mean rate constant of NTS (dark grey) and TS cleavage (light grey), and error bars show ± s.d. Dots show individual replicates.

Alignment of experimentally determined and predicted structures showed the Nuc-loop has divergent amino acid composition across the three Cas12a orthologues (Fig. 3A and Supplementary Figs. S8–S11). These loops contain a variety of charged and aromatic amino acid sidechains that could interact with nucleic acids. To disrupt these potential interactions, two general mutations were designed. Firstly, to remove any specific interactions by the Nuc-loop, but retain the steric bulk, the ‘head’ of the loop closest to the crRNA–TS heteroduplex was substituted for a flexible linker motif of repeating glycine-serine residues (Supplementary Figs. S8–S11 and Supplementary Table S3). This was termed the ‘FLX’ substitution (Supplementary Fig. S8–S11 and Supplementary Table S3). Secondly, to remove both steric bulk and any protein-nucleic acid interactions, the Nuc-loop was deleted—the ‘ΔLoop’ mutation (Supplementary Fig. S8–S11 and Supplementary Table S3).

Notably, AsCas12a has an additional insertion in the Nuc domain, not present in FnCas12a and LbCas12a, a motif we named ‘Nuc-loop 2′ (Fig. 3A and Supplementary Fig. S11). This loop does not extend towards the heteroduplex, instead, it sits on the surface of the Nuc and folds back towards the RuvC (Supplementary Fig. S11). Prolines often being crucial structural elements, we designed an alanine substitution of P1153 in Nuc-loop 2. Deletion of Nuc-loop 2 resulted in no soluble protein expression (data not shown), instead this motif was truncated and substituted with glycine-serine-glycine, a mutant we termed ‘FLX-2′ (Supplementary Table S3).

We first tested the thermostability of the Nuc-loop mutants. FnFLX and FnΔLoop had thermostability similar to WT enzyme, each at ∼48°C (Fig. 3B). LbFLX had similar thermostability to LbWT, at 42–43°C, while LbΔLoop was decreased at 37°C (Fig. 3C). AsFLX and AsP1153A were as stable as AsWT at 43–44°C, while AsFLX-2 was decreased at 39°C, and AsΔLoop had no detectable fluorescence peak (Fig. 3D).

Next, we characterized the *cis* cleavage kinetics of Nuc-loop mutants (Supplementary Figs S12–S15 and Supplementary Table S2). The FnFLX substitution mutant had slower *k*_NTS_ by 1.8×, while *k*_TS_ slightly increased by 1.3× (Fig. 3E). A more pronounced effect was observed in LbFLX, with *k*_NTS_ decreased by 3.9×, while *k*_TS_ increased by 2.3× (Fig. 3F). This suggests the Nuc-loop may restrain TS-loading for FnCas12a and LbCas12a, similar to the REC2 ‘gate’. In contrast, AsFLX exhibited a very specific effect on *k*_TS_, decreasing rates by 7×, and leaving *k*_NTS_ essentially unchanged (Fig. 3G). This indicates that AsCas12a, with already significantly slower TS cleavage rates, may use the Nuc-loop in a divergent manner.

Nuc-loop deletion had even more variable effects on DNA cleavage rates. FnΔLoop exhibited globally decreased *cis* cleavage compared to FnWT, *k*_NTS_ decreasing by 9.4× and *k*_TS_ decreasing by 4.3× (Fig. 3E). Interestingly, FnΔLoop only linearized ∼70% of the plasmid target, leaving a large ‘nicked’ fraction (Supplementary Fig. S12B and S16). This may indicate unstable target dsDNA binding. Despite its lesser thermostability, LbΔLoop showed similar *k*_NTS_ to LbWT, with *k*_TS_ slightly increased by 1.4× (Fig. 3F). AsΔLoop decreased *k*_NTS_ by 1.8×, and reduced *k*_TS_ 3.5× relative to AsWT (Fig. 3G). Both AsFLX and AsΔLoop showed incomplete cleavage of plasmid DNA, leaving ∼11% in the nicked state (Supplementary Fig. S14 and S16). This suggests the Nuc-loop of AsCas12a is important in completing TS cleavage.

Disruption of Nuc-loop 2 in AsCas12a decreased *cis* cleavage rates, despite its distance from any nucleic acids. P1153A substitution decreased *k*_NTS_ by 1.4×, while *k*_TS_ was unchanged (Fig. 3G). AsCas12a FLX-2 displayed very similar kinetics to AsΔLoop, with both *k*_NTS_ and *k*_TS_ 2.3× slower relative to AsWT (Fig. 3G). These latter two mutants being the least thermostable of the mutants generated, their decreased *cis* cleavage may stem from globally disrupted protein function, rather than from loss of specific Nuc-loop interactions.

Overall, these data suggest integrity of the Nuc-loop is important for the *cis* cleavage activity of Cas12a orthologues. To further characterize the role of Nuc-loops, we assayed their *trans* cleavage activity, their plasmid interference in *E. coli*, and their editing activities in mammalian cell lines.

**Figure 4.**
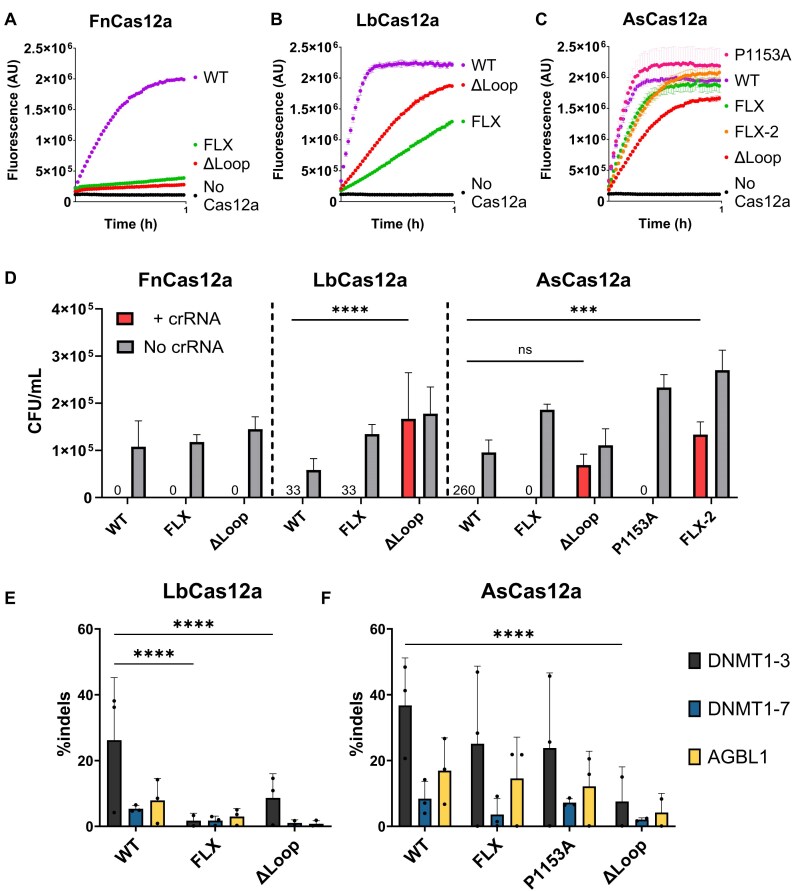
*Trans*cleavage curves with 23 nt spacer length crRNA, and full-length TS, for WT and Nuc-loop mutants of (**A**) FnCas12a, (**B**) LbCas12a, and (**C**) AsCas12a. Dots show mean, ± s.d. (**D**) Mean colony forming units per ml (error bars show s.d.), for ± crRNA conditions and Nuc-loop mutations as indicated. Statistical significance evaluated by two-way ANOVA with Tukey’s multiple comparison test. For LbCas12a, +crRNA condition; WT – ΔLoop *P* < .0001. For AsCas12a, +crRNA condition; WT – FLX-2, *P* = .0005. (**E**, **F**) Editing efficiency in HEK293T cell line. Mean percentage of insertions and deletions (indels) at the target site indicated (error bars show s.d.) for WT and Nuc-loop mutants of (**E**) LbCas12a and (**F**) AsCas12a. Statistical significance evaluated by two-way ANOVA with Tukey’s multiple comparison test. For DNMT1–3 site, LbCas12a; WT-FLX *P* < .0001, WT-ΔLoop *P* < 0.0001. For DNMT1–3 site, AsCas12a; WT-ΔLoop *P* < .0001.

As with REC2 ‘gate’ mutants, the *trans* cleavage activity was tested with four different combinations of crRNA and TS DNA. Unlike the REC2 mutants, Nuc-loop mutants did not show consistent patterns of substrate-dependant activity (Supplementary Figs. S17–S20). For clarity, only *trans* cleavage reactions with the 23 nt crRNA and full-length TS are shown in Fig. 4.

Compared to FnWT, the FnFLX and FnΔLoop mutants had greatly decreased *trans* cleavage (Fig. 4A). To a lesser extent, LbFLX and LbΔLoop mutants had reduced *trans* cleavage relative to LbWT (Fig. 4B). AsP1153A showed similar *trans* cleavage to AsWT, while AsFLX, AsFLX-2, and AsΔLoop had moderately decreased *trans* cleavage activity (Fig. 4C). These data indicate the Nuc-loop plays a key role in the *trans* cleavage activity of FnCas12a. For LbCas12a and AsCas12a, Nuc-loop mutation decreases *trans* cleavage, although not to the magnitude of FnCas12a mutants.

Next, we tested the function of these Nuc-loop mutants for their ability to interfere with plasmid transformation in *E. coli*. Briefly, plasmids encoding Cas12a were transformed into *E. coli* strains harbouring either an empty vector (no crRNA) or a crRNA-encoding vector (+crRNA) that directed Cas12a to cleave a third ‘target’ vector. FnFLX and FnΔLoop showed as robust plasmid interference as FnWT, with no colonies observed for the ‘+ crRNA’ condition (Fig. 4D). LbWT and LbFLX also showed strong plasmid interference, but LbΔLoop showed a loss of activity, with CFU/mL counts similar to the ‘no crRNA’ transformations (Fig. 4D). AsWT, AsFLX, and AsP1153A had robust plasmid interference, while AsΔLoop and AsFLX-2 lost plasmid interference activity (Fig. 4D). Notably, it is the least thermostable Cas12a mutants (LbΔLoop, AsΔLoop, and AsFLX-2) that displayed the weakest plasmid interference, highlighting the importance of protein integrity in this assay.

As these Nuc-loop mutants had novel effects on target dsDNA cleavage *in vitro*, we aimed to assess their gene editing efficiency. Cas12a ribonucleoprotein complexes were electroporated into human cell lines, and insertions and deletions (indels) at target sites were quantified by high-throughput sequencing. Cas12a mutants were tested in HEK293T, A549, and Jurkat cell lines (Fig. 4E and F; Supplementary Figs. S21–S24), for clarity, only HEK293T editing is displayed in Fig. 4. We observed lower editing efficiency for FnCas12a compared to AsCas12a and LbCas12a (Supplementary Fig. S21), in agreement with previous works [5, 6]. We therefore decided not to pursue further human cell line editing with FnCas12a mutants.

Despite the robust *E. coli* plasmid interference of LbFLX, it was significantly less active than LbWT in genome editing (Fig. 4E). Expected from its lower activity in *E. coli*, LbΔLoop exhibited much lower editing compared to LbWT (Fig. 4E and Supplementary Figs. S22–S24). AsFLX and AsP1153A displayed a similar indel rate to AsWT, across cell lines and target sites (Fig. 4E and Supplementary Figs. S22–S24). AsΔLoop showed consistently decreased editing efficiencies, as expected from its weak interference in *E. coli*.

Given their slower cleavage of target dsDNA *in vitro*, we aimed to test if AsFLX and AsP1153A exhibited less off-target editing than AsWT. The most frequent off-targets for the DNMT1–3, DNMT1–7, and AGBL1 sites were derived from [6], and indels quantified by high-throughput sequencing (Supplementary Figs. S25–S27). Off-target editing was generally low for all nucleases (Supplementary Figs. S25–S27). We observed sporadic increases in off-target indels compared to the No Cas12a control, but no nuclease showed a consistent pattern of significantly different off-target edits across all off-targets and cell lines tested (Supplementary Figs. S25–S27).

Overall, the Nuc-loop can play a critical role in the *in vitro* and *in vivo* function of Cas12a, but this varies considerably between orthologues. Strikingly, although FnΔLoop was the most disrupted of all the FnCas12a mutants generated, it still retained *in vitro* TS cleavage rates 9× faster than wild-type AsCas12a. To understand this difference, we looked for more global drivers of catalytic function.

### Molecular dynamics simulations reveal distinct probabilities of ‘clamping’ between Cas12a orthologues

Recent works have demonstrated the importance of dynamic conformational changes in catalysis by Cas12a [14, 18, 24, 28–31, 69]. On binding a matching DNA target, stable contacts are formed between the crRNA:TS heteroduplex and the REC2 domain [14, 18, 70]. These interactions constrain the flexibility of the REC2 [18]. This allows the BH domain to ‘dock’ with the REC2 domain and form contacts that ‘open’ the RuvC-lid, thus allosterically activating DNA cleavage [18, 21–23]. In the process of sequentially cleaving NTS and TS, inward motions of REC2—Nuc domains have been observed in single-molecule FRET experiments and predicted in MD simulations [14, 24, 28–31].

The inward ‘clamping’ motions between REC2 and Nuc are correlated with TS cleavage, in which an especially high FRET state is seen immediately before TS cleavage and substrate release [28, 30, 31]. Given the disparities in *k*_TS_ we observe, we wished to compare REC2—Nuc dynamics between wild-type Cas12a orthologues. Furthermore, as the Nuc-loop extends towards the REC2 domain, we reasoned it may make contact with the REC2 in the dynamic motions of DNA cleavage. To test this, we performed μs-length classical MD simulations of Cas12a orthologues. We employed structures of Cas12a in their ternary state, i.e. in complex with crRNA and target DNA.

**Figure 5.**
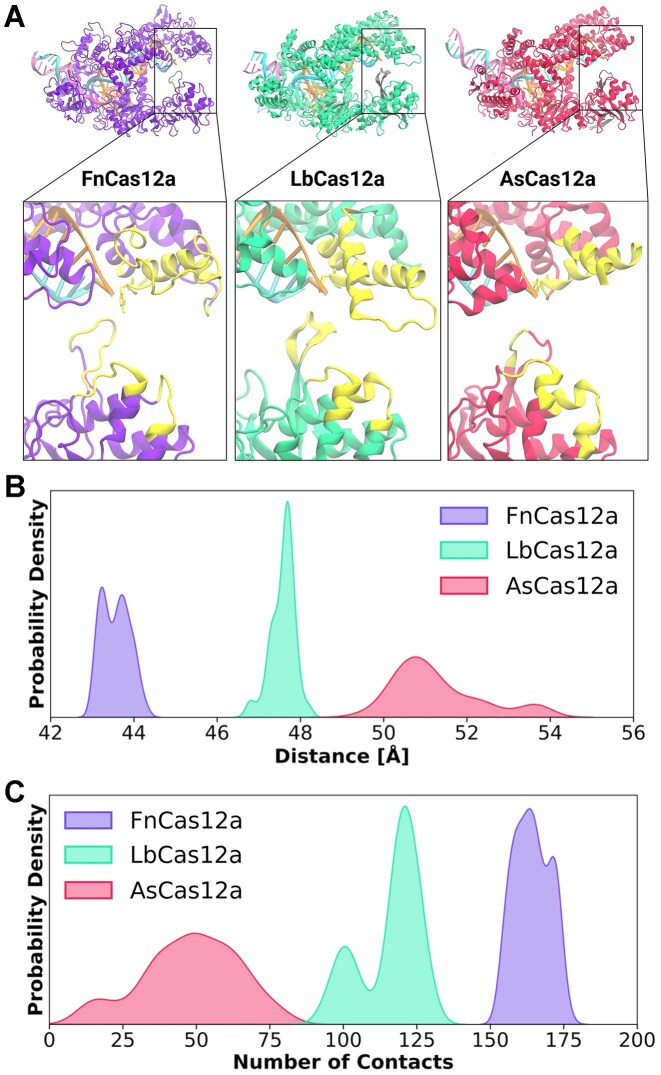
MD simulations of Cas12a ternary complexes. Microsecond-long simulations were performed on ternary complexes of FnCas12a (6GTG), LbCas12a (5XUS), and AsCas12a (5B43). (**A**) Visualization of residues involved in REC2-Nuc contacts (yellow), overlaying Fn Cas12a (6GTG, purple), Lb Cas12a (AF2 prediction, bright teal), and As Cas12a (8SFQ, red). Shown are the crRNA (orange) and target strand (cyan). Kernel density estimation plots of probability density for (**B**) distance between REC2 and Nuc and (**C**) probability density of contacts between REC2 and Nuc.

These simulations detailed the residues involved in the REC2-Nuc contacts (Fig. 5A, enumerated in Supplementary Table S4). Notable amongst these contacts is the Nuc-loop. Additionally, the PAM-distal tip of the Nuc domain also makes contact with the REC2. These Nuc regions interact with the REC2 domain in the most distal regions, where the ‘gate’ residue is located. Notably, extensive Nuc-loop and heteroduplex interactions were also seen in recent high-resolution simulations of FnCas12a cleaving the TS [24].

These data show the three Cas12a orthologues have distinctly different conformational distributions. FnCas12a has probability density centred at 43–44 Å, LbCas12a at 47–48 Å, and AsCas12a has a broad distribution from 50–54 Å (Fig. 5B, Supplementary Fig. S28). As the closed conformation is thought to be particularly important for TS cleavage [24, 28, 30, 31], it is intriguing to note these distributions line up with observed rates of *k*_TS_ between Cas12a orthologues.

A cause, or consequence, of these distributions are the protein–protein contacts between the REC2 and Nuc (Fig. 5C and Supplementary Fig. S29). The probability of the number of contacts between the REC2 and Nuc was quantified, and these show that FnCas12a has a high number of REC2-Nuc contacts, peaking at ∼170 (Fig. 5C). LbCas12a has two distinct peaks at ∼100 and ∼125 contacts, perhaps indicating two distinct conformations (Fig. 5C). Strikingly, AsCas12a ranged from zero to almost one hundred contacts, peaking at ∼50 (Fig. 5C).

In light of recent structural data characterizing the conformation changes of the REC2 domain of AsCas12a in DNA cleavage [18], these REC2–Nuc dynamics are significant. REC2 flexibility was observed throughout the early stages of target dsDNA binding, with stable ‘docking’ between REC2 and BH domain only occurring at a complete 20 bp R-loop [18]. These simulations suggest Cas12a orthologues may differ in their conformational flexibility, even at complete R-loop formation. More dynamic REC2–Nuc motions may decrease BH-docking and reduce allosteric activation of the RuvC. By enumerating the residues involved in REC2–Nuc ‘pinching’ motions, we find an interplay between the Nuc domain, Nuc-loop, and REC2 domain.

## Discussion

We explored what drives the difference in function between Cas12a orthologues, using a combination of *in vitro* DNA cleavage assays, *in vivo* plasmid interference, genome editing, and *in silico* simulations. Our results show trade-offs between NTS, TS, and *trans* cleavage, which may be driven by dynamic REC2–Nuc interactions.

### Kinetic comparison of wild-type Cas12a orthologues

We observed large differences in *cis* cleavage kinetics between Cas12a orthologues. These differences in strand cleavage kinetics are important, given a growing body of evidence that the kinetics of R-loop formation and DNA cleavage drive the target specificity of Cas12a nucleases [20, 69, 71, 72].

For LbCas12a and AsCas12a, the values of *k*_NTS_ and *k*_TS_ were comparable to previously published values [18, 20, 25–27] (Supplementary Table S1). The rates for FnCas12a were over 10x faster than a previous study [23], which was itself over 5x faster than another report [14] (Supplementary Table S1). Notably, these studies both use half the Mg^2+^ ion concentration than herein (5 versus 10 mM MgCl_2_), which has been shown to decrease both DNA binding and *cis* cleavage rates for AsCas12a [20, 28]. Furthermore, both previous kinetic studies for FnCas12a used short linear dsDNA substrates [14, 23]. Previous work has shown negatively supercoiled DNA substrates accelerate R-loop formation for LbCas12a compared to the unconstrained topology of linear DNA substrates [27]. Faster cleavage of plasmid DNA vs short oligonucleotides has also been observed for FnCas12a [23]. The rapid plasmid DNA cleavage by FnCas12a in this study suggests it also has more rapid R-loop formation with negatively supercoiled DNA substrates, in contrast to AsCas12a, which has very similar *cis* cleavage kinetics between substrates [18, 20, 25] (Supplementary Table S1).

The R-loop formation of AsCas12a has been studied in detail and is thought to occur with minimal contribution from the REC domain [18, 20]. The faster and more torque-sensitive R-loop formation by FnCas12a and LbCas12a may indicate divergent Cas12a-heteroduplex interactions in target dsDNA recognition. Supporting this are high-throughput mismatch studies on plasmid DNA targets, which show that AsCas12a is much more specific than LbCas12a and FnCas12a [71]. Critically, the kinetics of strand cleavage rates influence targeting specificity in a biological setting [72]. Our comparison of wild-type FnCas12a, LbCas12a, and AsCas12a provides a kinetic explanation for these observed differences in specificity.

### REC2 and Nuc-loop interactions modulate strand cleavage kinetics

**Figure 6.**
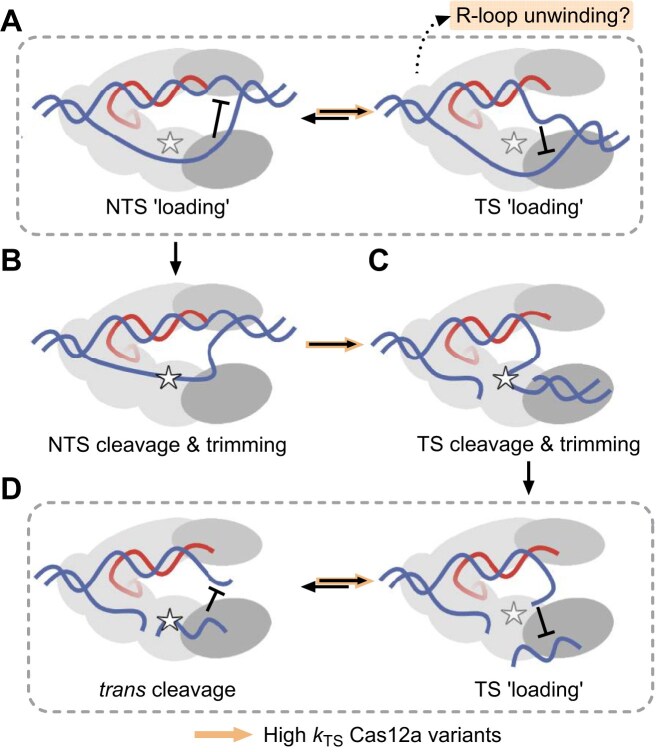
Proposed model of trade-offs between high TS cleavage rates and NTS and *trans* cleavage. (**A**) At complete R-loop formation, the NTS and TS compete for binding Nuc residues near the RuvC. Putative instability of the TS-loading state for high *k*_TS_ Cas12a variants (AsW382A, LbFLX, LbW55A, FnWT, FnFLX, and FnY410A), resulting in incomplete target dsDNA cleavage. (**B**) Successful NTS loading precedes NTS cleavage and trimming. This is slowed by more frequent TS loading. (**C**) TS cleavage and trimming, which is accelerated for high *k*_TS_ Cas12a nucleases. (**D**) Loading of the trimmed, PAM proximal TS fragment competes with *trans* ssDNA substrates for binding at the Nuc. High *k*_TS_ Cas12a variants have slower *trans* cleavage as a result.

We replicated and expanded on previous work studying REC2 mutations [15, 26]. We observed that alanine substitution of the REC2 ‘gate’ resulted in consistent increases in *k*_TS_ across Cas12a orthologues, to the detriment of *k*_NTS_ (Fig. 2A–C). With rapid TS cleaving REC2 mutants, we observed slower *trans* cleavage when activated by full-length vs truncated TS for FnCas12a and AsCas12a (Fig. 2D–G). LbW355A had slower *trans* cleavage than WT for all combinations of crRNA and TS (Fig. 2D–G). However, a recent study comparing LbCas12a WT and W355A tested a target strand truncated to 19 nt, and found this resulted in a four-fold increase in *trans* cleavage rate compared to a full-length TS [67]. Despite LbCas12a trimming the TS to positions 22–23 relative to the PAM [26], similar to FnCas12a and AsCas12a [22, 25], it would appear a 20 nt-length target strand is sufficient to sterically inhibit loading of *trans-*ssDNA substrates. Overall, this suggests TS-loading of the PAM-proximal fragment can sterically hinder *trans* ssDNA substrates from cleavage (Fig. 6).

This agrees with the observation that excess ssDNA can slow TS cleavage [28]. A wealth of biochemical evidence has established the sequential order of NTS, TS, and then *trans* cleavage [9, 13, 14, 25–27]. This obligatory sequential mechanism is driven by occlusion at the narrow active site cleft, although NTS and TS are coordinated by larger networks of interactions across the RuvC and Nuc domains [4, 13, 14, 18, 25]. We suggest that the concomitant decrease of *k*_NTS_ with increased *k*_TS_ is caused by competitive binding between NTS and TS near the active site (Fig. 6). It is probable that non-productive TS-loading conformations can occur before the NTS is cleaved, given that dynamic unwinding of the TS from crRNA is observed in the absence of DNA cleavage [26]. Similarly, we suggest reduced *trans* cleavage with REC2 mutants is driven by competition between TS and *trans* ssDNA substrates. Notably, the TS is already cleaved and trimmed to its maximum extent (after a 45 min incubation), suggesting steric hindrance is not in the active site itself but at nearby residues (Fig. 6).

Given REC2 mutants have faster TS cleavage than WT, it has been questioned why this aromatic interaction is conserved across the Cas12a family [26]. Although we observed no defect in thermostability or in *E. coli* plasmid interference (Supplementary Fig. S7), these mutants had incomplete *cis* cleavage compared to WT (Supplementary Fig. S5). Others have suggested instability of mutant proteins as a cause of incomplete DNA cleavage, on the basis of protein degradation visible on SDS–PAGE [26]. We observe similar protein degradation products in SDS–PAGE (Supplementary Figs. S30–S31), which do not correspond to lessened thermostability (Supplementary Fig. S7C).

We suggest this incomplete *cis* cleavage is a symptom of lessened R-loop stability. Incomplete *cis* cleavage has been previously observed for LbW355A, but only when using a 20 nt spacer crRNA (∼20% of supercoiled target dsDNA uncut) [26]. In our assays with a 23 nt spacer crRNA we observed ∼12% of supercoiled DNA left uncut for LbW355A and FnY410A, significantly more than seen for WT nucleases and AsW382A (∼5%) (Supplementary Fig. S5).

Similar to LbW355A, the LbFLX mutant increased *k*_TS_ and decreased *k*_NTS_, with ∼12% of target plasmid uncut (Supplementary Fig. S16). LbFLX had showed very different activity between *E. coli* plasmid interference and human cell line editing, with significantly reduced activity in the latter (Fig. 4D and E). We suggest that FLX substitution in LbCas12a weakens critical Nuc-loop target dsDNA interactions, resulting in disrupted strand cleavage kinetics similar to REC2 mutants (*k*_TS_ > *k*_NTS_) and lower editing activity in human cell lines. This may have parallels to the lower of gene editing observed for AsW382A [15].

For AsCas12a, AsFLX had a specific effect on *k*_TS_ only (Fig. 3G). This mutant retained wild-type levels of activity in *E.coli* plasmid interference and in editing human cell lines (Fig. 4D and F). We propose this mutation minimally disrupts the R-loop formation process of AsCas12a. Likewise, but more trivally, the AsP1153A mutant was not significantly different to wild-type AsCas12a. LbΔLoop, AsΔLoop, and AsFLX-2 mutants had relatively active *cis* and *trans* cleavage at 30°C (Figs 3F and G and 4B and C) and poor activity in *E. coli* and/or human cell line editing at 37°C (Fig. 4E and F). Any interpretation regarding the effect of Nuc-loop disruption on their function is confounded by their globally lower stability (Fig. 3C and D).

The FnΔLoop mutant showed partial *cis* cleavage, leaving ∼20% of the target plasmid in the nicked state (Supplementary Fig. S16). This activity is not likely to be caused by non-specific nicking by excess nuclease present in the single-turnover conditions, as this mutant is minimally *trans-*active (Fig. 4A). Unlike the deletion mutants of LbCas12a and AsCas12a, this mutant displayed wild-type levels of thermostability and plasmid interference in *E. coli* (Figs 3B and 4D). Similar to REC2 mutants, the incomplete *cis* cleavage of FnΔLoop did not impair its plasmid interference ability (Fig. 4D). We suggest the FnΔLoop has incomplete target dsDNA cleavage through R-loop collapse and target DNA dissociation, caused by loss of critical Nuc-loop interactions. Partial cleavage of target dsDNA (i.e. NTS nicking only) has also been observed at mismatched target sites [71, 73]. These studies use negatively supercoiled target plasmids [71, 73], a substrate topology that can allow rapid R-loop formation [27]. Mismatches between crRNA and TS decrease R-loop stability and can lead to dissociation of Cas12a [20]. In target-dependent nicking, Cas12a appears to form stable-enough ternary complexes to permit NTS cleavage but dissociate before TS cleavage [71, 73]. Notably, it was shown each orthologue has different levels of incomplete target cleavage, where FnCas12a > LbCas12a > AsCas12a [71], perhaps indicative of general R-loop stability across matched and mismatched target sites.

In this study and elsewhere, it is notable that plasmid interference in *E. coli* does not correlate to activity in human cell line editing [5]. We propose the mutations causing faster *k*_TS_ may decrease the stability of target dsDNA binding and cause loss of function in gene editing. As the conformation of TS-loading requires unstacking of the REC2 gate [26] and downstream unwinding of the crRNA:TS heteroduplex [25], we propose the TS-loading conformation of Cas12a must be tightly regulated to avoid R-loop instability. Kinetic and biophysical studies have shown R-loop formation is reversible before NTS cleavage [20, 26]. Loss of REC and Nuc-loop interactions may license premature TS-loading, an unstable state that competes with NTS loading near the RuvC. In this way, we speculate that high *k*_TS_ variants of Cas12a (AsW382A, LbFLX, LbW55A, FnWT, FnFLX, and FnY410A) also have weaker initial target dsDNA binding, causing incomplete target cleavage (Fig. 6).

In a similar vein, two biophysical studies have shown FnCas12a has weaker target dsDNA interactions than LbCas12a and AsCas12a [19, 69]. An smFRET study of target dsDNA interrogation determined FnCas12a has a lower *K*_D_ compared to LbCas12a and AsCas12a, driven by a slow *k*_ON_ [19]. In target dsDNA search, FnCas12a has notably slower one-dimensional diffusion rates than LbCas12a and AsCas12a [69]. This latter work showed diffusion relies on an alpha helix in the REC2, rich in positively charged residues, alanine substitution of which reduced diffusion rates and genome editing in HEK293T cells [69]. This suggests the weak target dsDNA search and binding of FnCas12a is not problematic in *E. coli* plasmid interference but critical in human cell line editing.

### Molecular dynamics simulations reveal inter-orthologue differences in dynamic states

MD simulations showed the REC2 and Nuc domain make numerous contacts in their dynamic motions, the REC2 ‘gate’ and Nuc-loop notably amongst them (Fig. 5A and Supplementary Table S4). This lends credence to the notion that the Nuc-loop aids the TS to traverse the distance between REC2 ‘gate’ and RuvC. The predicted distance distributions, FnCas12a < LbCas12a < AsCas12a, fit well with observed TS cleavage rates (Figs 1H and 5B). Given the importance of the close REC2–Nuc conformation in TS cleavage [14, 24, 28–31], we propose this is a major driver of different TS cleavage rates between Cas12a orthologues.

Stable ‘docking’ of the REC2 to the BH domain upon 20 bp R-loop formation has recently been described as critical to the allosteric activation of the RuvC [18, 21–23]. This reduction in conformational flexibility of the REC2 first licenses NTS cleavage, TS cleavage, then *trans* cleavage [18]. MD performed on ternary complexes suggests Cas12a orthologues may differ substantially in their conformational flexibility when bound to a 20 bp R-loop. The lesser flexibility of FnCas12a may drive its rapid *k*_NTS_ and *k*_TS_, which in turn slows its *trans* cleavage of ssDNA through steric hindrance near the RuvC. The greater flexibility of AsCas12a may drive its slow *k*_NTS_ and *k*_TS_. The hypothesis of TS-loading as inhibitory to *trans* cleavage would predict higher *trans* cleavage rates for AsCas12a than LbCas12a, due to its lower *k*_TS_. However, we see consistently higher trans cleavage by LbCas12a. We propose that the low *k*_TS_ of AsCas12a is driven by the higher conformational flexibility of its REC2 domain, as observed in larger REC2-Nuc distances in our MD simulations, and lack of resolution in cryo-EM studies [18]. This high flexibility likely reduces *trans* cleavage by weakening the allosteric activation of the RuvC, which requires stable contacts between the BH and REC2 domains. The intermediate REC2-Nuc distances of LbCas12a may result in a more optimal trade-off between TS and *trans* cleavage than the conformations of AsCas12a and FnCas12a.

In total, we propose that REC2-Nuc dynamics are important drivers of the catalytic activation of Cas12a orthologues.

## Conclusions

What makes an effective Cas12a? We sought to compare the properties of three Cas12a orthologues *in vitro, in vivo*, and *in silico*, and to integrate these findings with the broader Cas12a literature to answer this question. We found that although FnCas12a has more rapid *cis* cleavage than LbCas12a and AsCas12a, the rapid TS-loading may sterically inhibit its *trans* cleavage. We replicated findings that REC2 mutants increase *k*_TS_ and expand this to suggest the unrestrained TS-loading may sterically slow NTS-loading and *trans* cleavage.

We explored the role of the Nuc-loop in the function of Cas12a orthologues. Deletion of the Nuc-loop was destabilizing for LbCas12a and AsCas12a, limiting the conclusions we can draw. Nonetheless, noting the increased *k*_TS_ of FnFLX and LbFLX mutants, we propose the Nuc-loop helps to restrain TS-loading, perhaps in cooperation with the REC2 gate. However, the decreased *k*_TS_ of AsFLX would indicate this is not universal. Mutations that increase TS-loading appear detrimental to function in human cell line editing but not in plasmid interference. Observing incomplete target dsDNA cleavage *in vitro*, we propose the TS-loading conformation of these nucleases is unstable, resulting in weaker target dsDNA binding.

MD simulations predicted numerous interactions between REC2, Nuc-loop, and Nuc domain, and further suggested the conformational flexibility of Cas12a ternary complexes differs greatly between orthologues. The more compact conformation assumed by FnCas12a may drive its very high TS cleavage rates and reduce its ability to stably bind target dsDNA.

Overall, we propose there exists a trade-off between stable target dsDNA binding and rapid target cleavage. Naturally occurring Cas12a orthologues studied herein appear to occupy different places in this trade-off, with FnCas12a optimized for rapid *cis* cleavage and AsCas12a for slow but stable *cis* cleavage. This has implications for their *in vitro* application in molecular diagnostics, with LbCas12a seeming to occupy a more optimal *cis/trans* cleavage trade-off. We propose that future engineering efforts explore this trade-off in the search for improved Cas12a nucleases.

## Supplementary Material

gkaf988_Supplemental_File

## Data Availability

Data from high-throughput sequencing of on and off-target genome editing have been deposited with the National Center for Biotechnology Information Sequence Read Archive under BioProject ID PRJNA1281281. All other datasets are available on request.

## Notes

Present address: Department of Systems Biology, Columbia University Irving Medical Center, New York, NY 10032, United States
