# Supplementary material for: CRISPR-Cas12a REC2–Nuc interactions drive target-strand cleavage and constrain trans cleavage
Source: Nucleic Acids Res. 2025 Oct 8;53(18):gkaf988. doi: 10.1093/nar/gkaf988 (PMC12507515; doi:10.1093/nar/gkaf988)
Supplement: gkaf988_Supplemental_File [file gkaf988_supplemental_file.pdf]

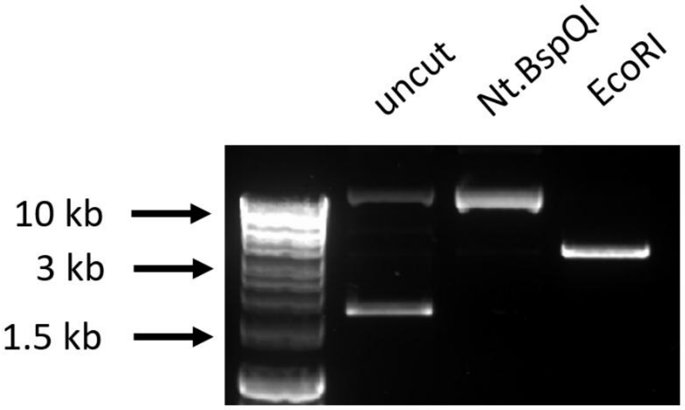

Figure S1: *Changes in target plasmid topology with nicking by Nt.BspQI, and linearisation with EcoRI.*

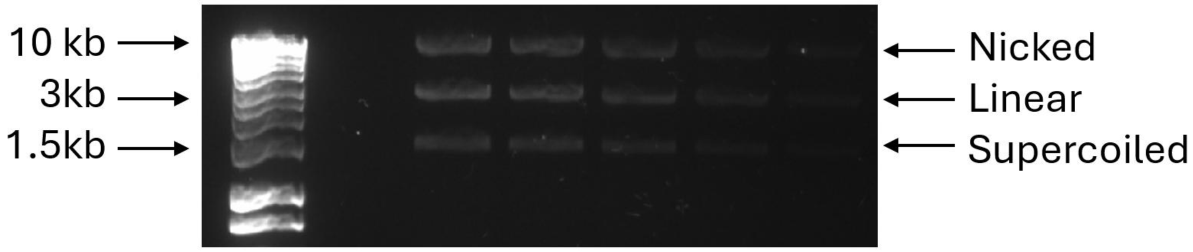

Figure S2: *Example dilution series of equal parts nicked, linear, and supercoiled plasmid DNA, for calibration of GelRed binding. Nicked fraction generated by digestion with Nt.BspQI, linear fraction with EcoRI. Three replicate dilution series were performed.*

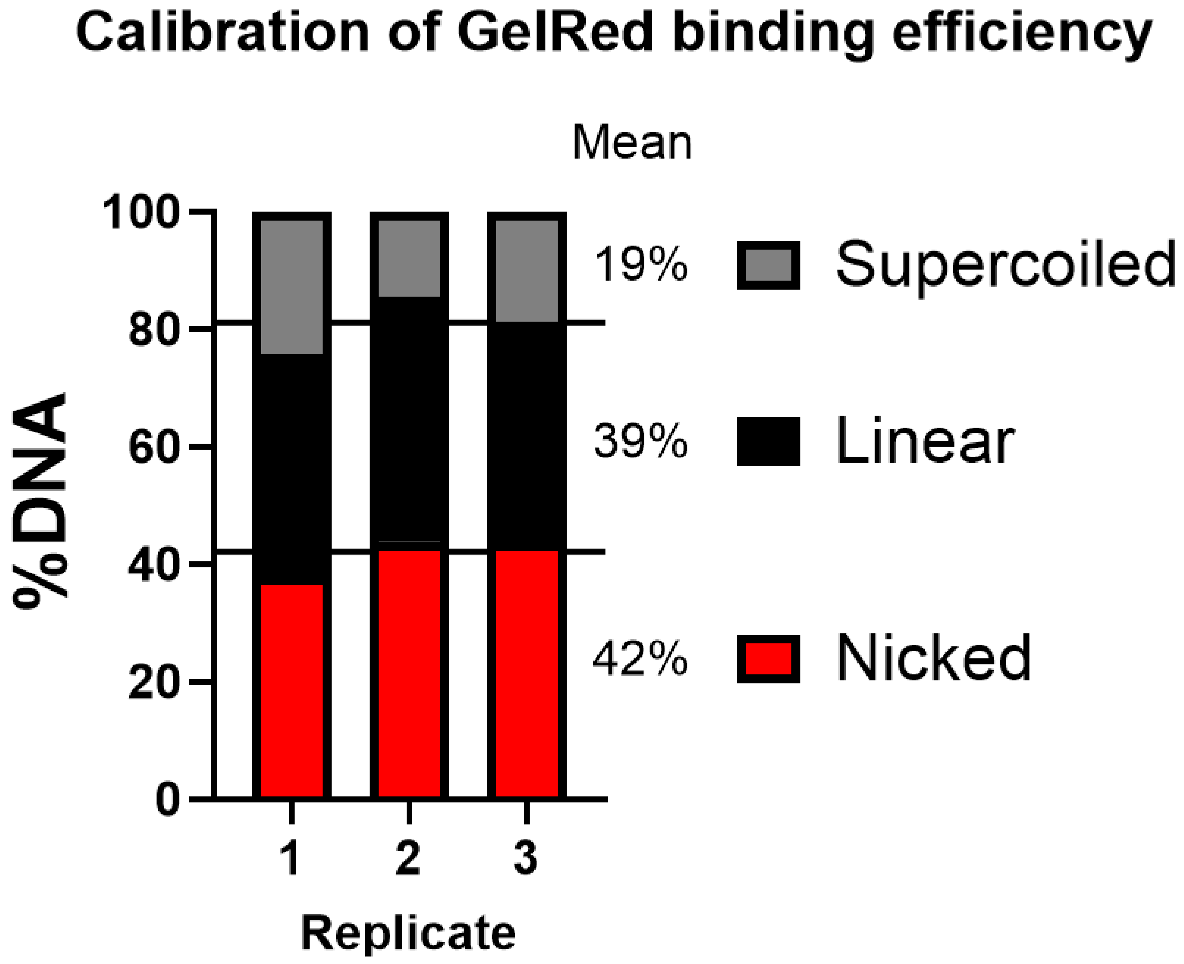

Figure S3: *Quantification of GelRed binding to equal quantities of nicked, linear, and supercoiled DNA. Bars show mean DNA percentage across a single dilution series, of supercoiled (grey), linear (black) and nicked DNA (red). Text shows mean percentage across the three replicates.*

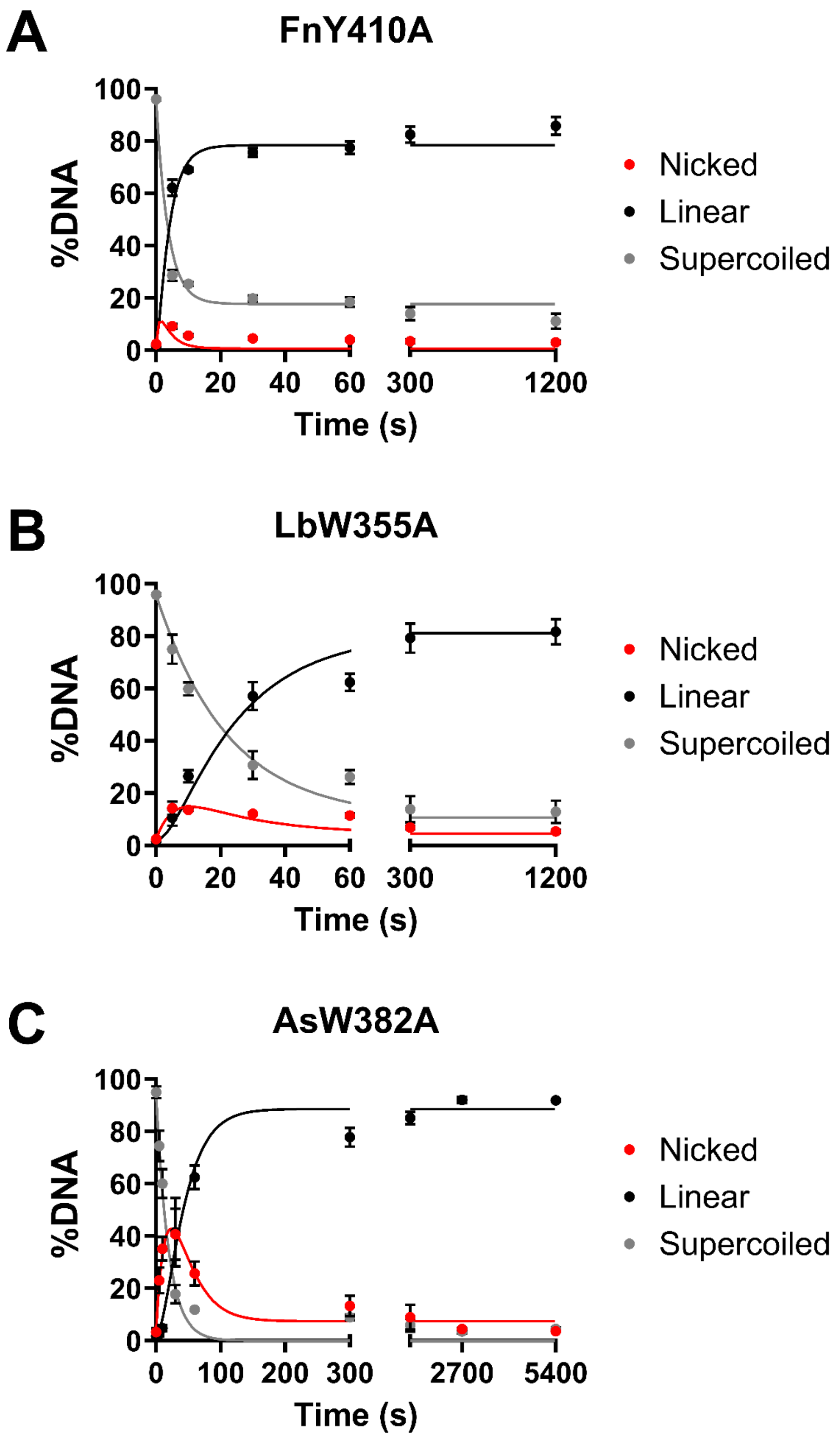

FigureS4: *Quantification of DNA fractions over time, with Cas12a mutant indicated. Line shows fit of mean rate constants, dots show mean %DNA, error bars ± s.d..*

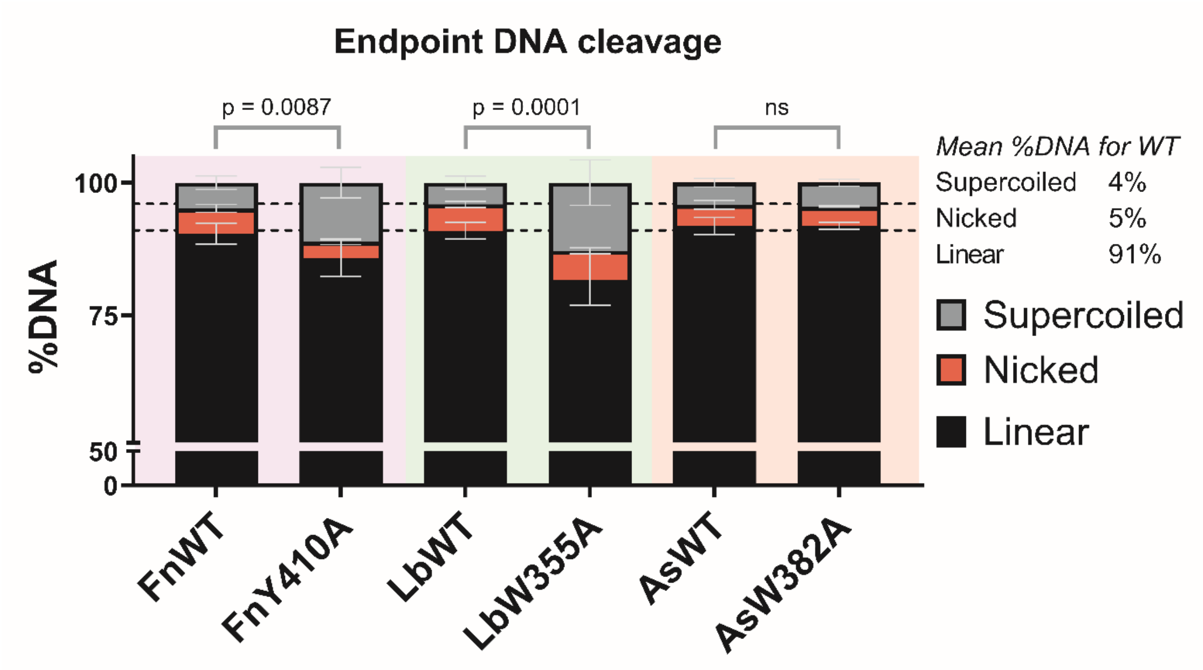

FigureS5: *Comparison of DNA fractions at endpoint of cis cleavage assays. Complete cis cleavage results in linearised plasmid DNA (black), partial cleavage leaves nicked plasmid products (red), and some fraction of target plasmid remains uncut and negatively supercoiled (grey). Bars show mean, error bars ± s.d. Dotted horizontal lines show the mean of DNA fraction across WT FnCas12a, LbCas12a, and AsCas12a. Grey brackets show statistical comparisons of supercoiled (uncut) DNA at endpoint of reaction. Statistical significance evaluated by two-way ANOVA with Tukey's multiple comparison test.*

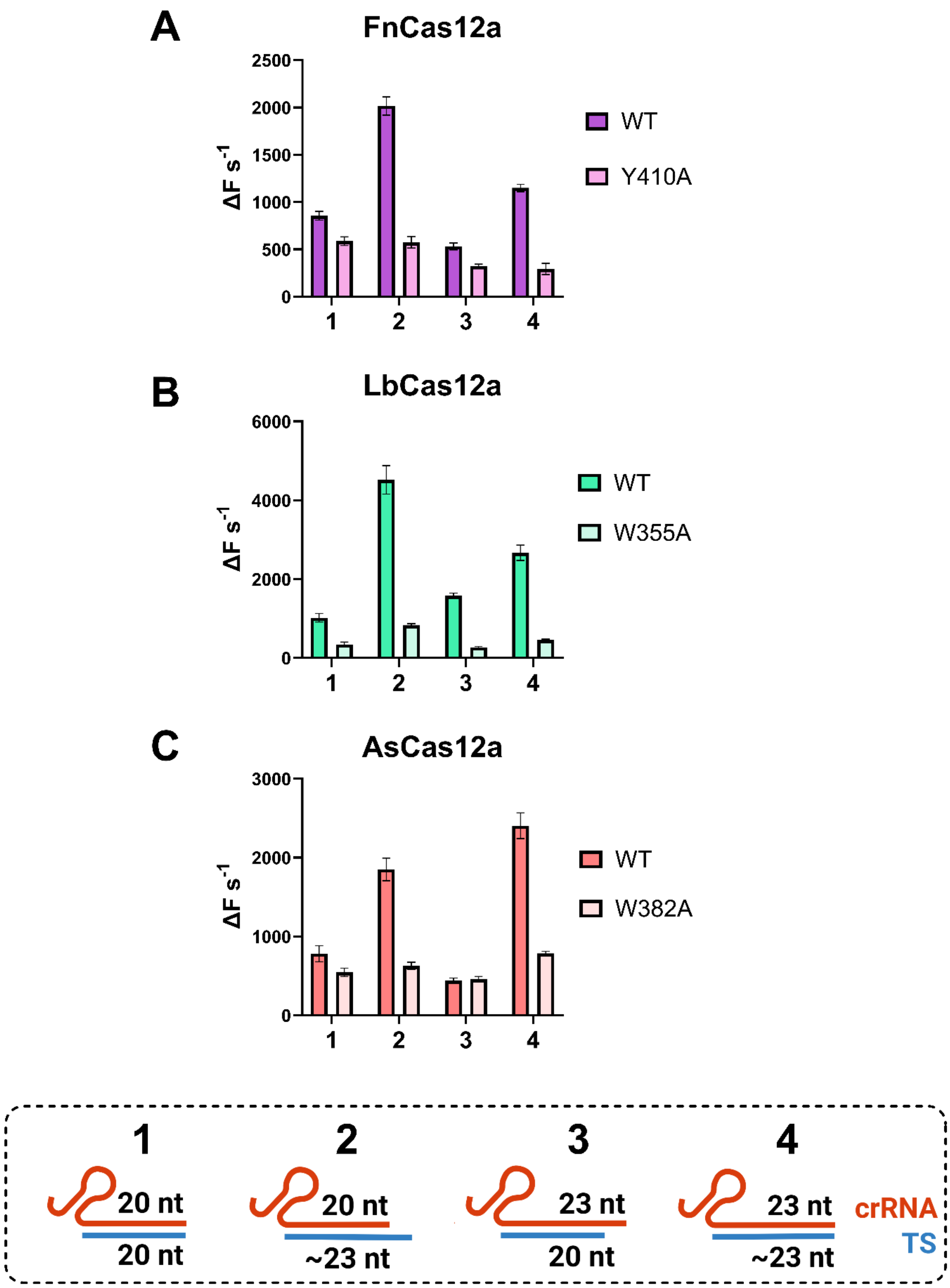

FigureS6: *Quantification of slope of first 300s of trans cleavage curves, as determined by linear regression of each replicate. Bar shows mean, error bars show 95% confidence intervals. Calculated in Graphpad Prism 10.*

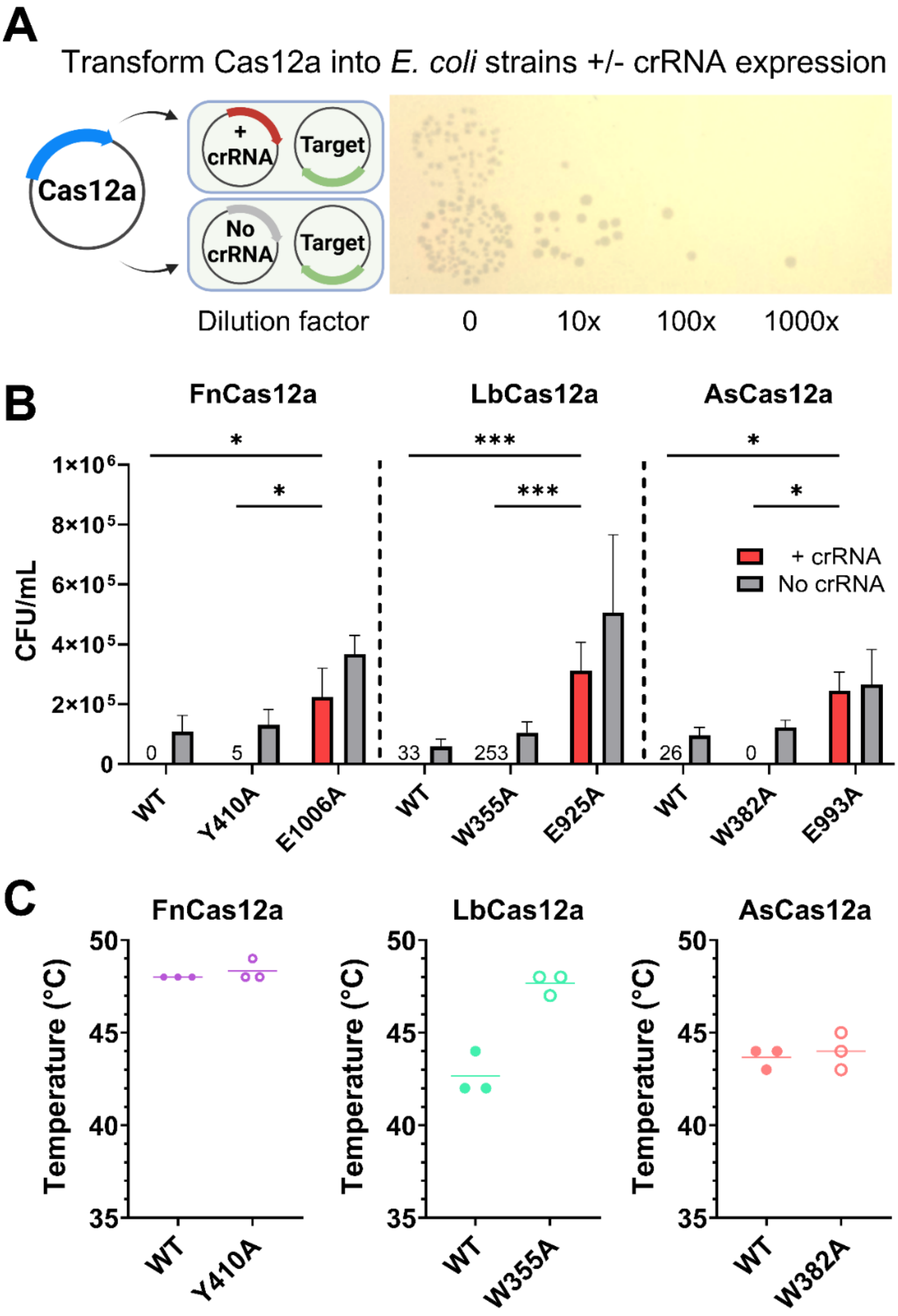

Figure S7: *(A) Outline of plasmid interference assay, image shows example transformation. (B) Mean colony forming units per mL (error bars ± s.d.), for +/- crRNA conditions, Cas12a as indicated. Statistical significance evaluated by two-way ANOVA with Tukey's multiple comparison test. FnCas12a, +crRNA condition; WT-E1006A p =0.0379, Y410A-E1006A p =0.0380. LbCas12a, +crRNA condition; WT-E925A p =0.0010, W355A-E925A p=0.0010. AsCas12a, +crRNA condition; WT-E993A p =0.0169, W382A-E993A p =0.0167. (C) Thermostability assay, melting point defined as fluorescence peak, line shows mean of three replicates.*

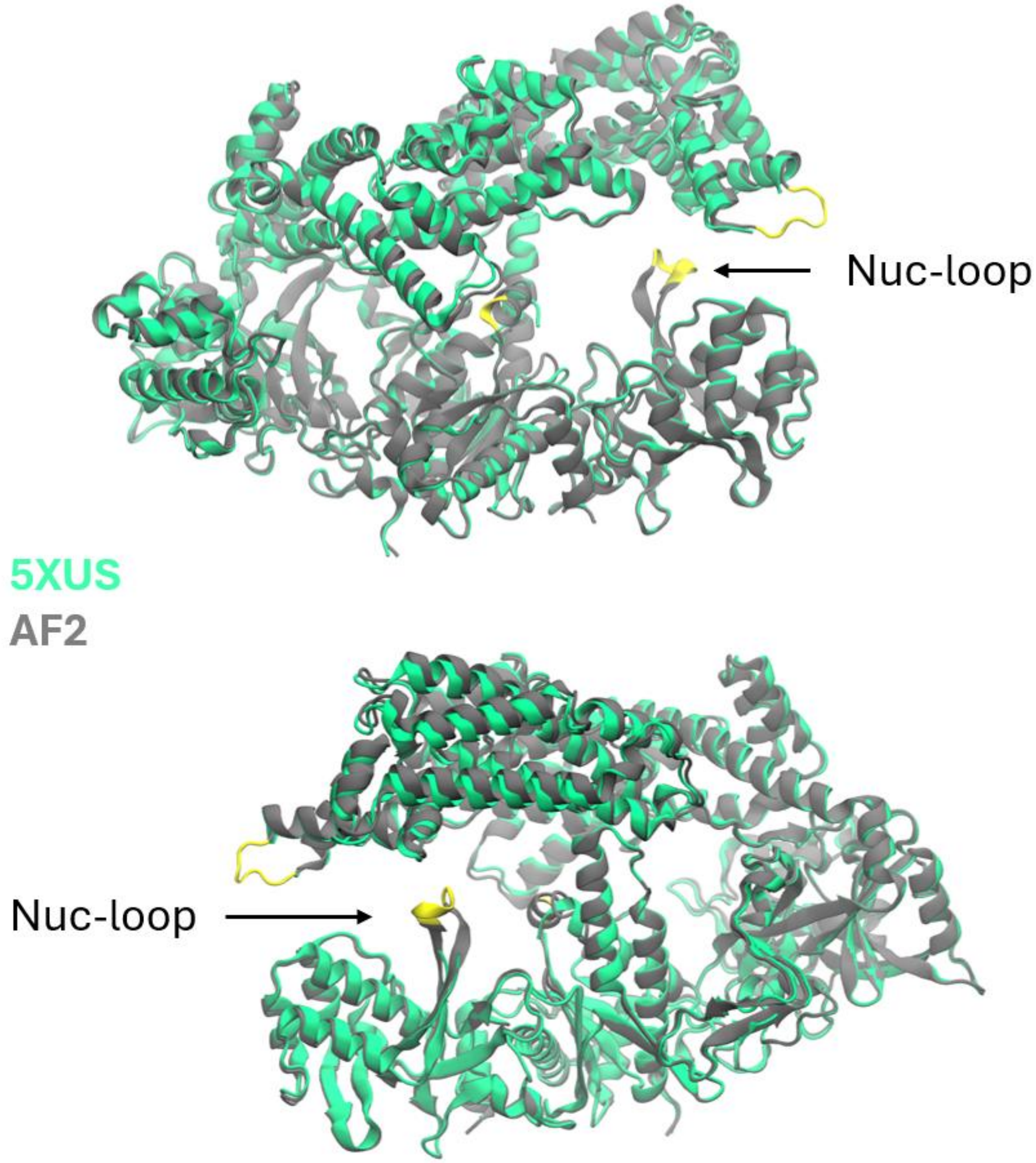

Figure S8: *Structural alignment (STAMP) of LbCas12a, crystal structure 5XUS (green) and AF2 model (grey – accessed from https://alphafold.ebi.ac.uk/entry/A0A182DWE3), with pLDDT <70 (yellow).*

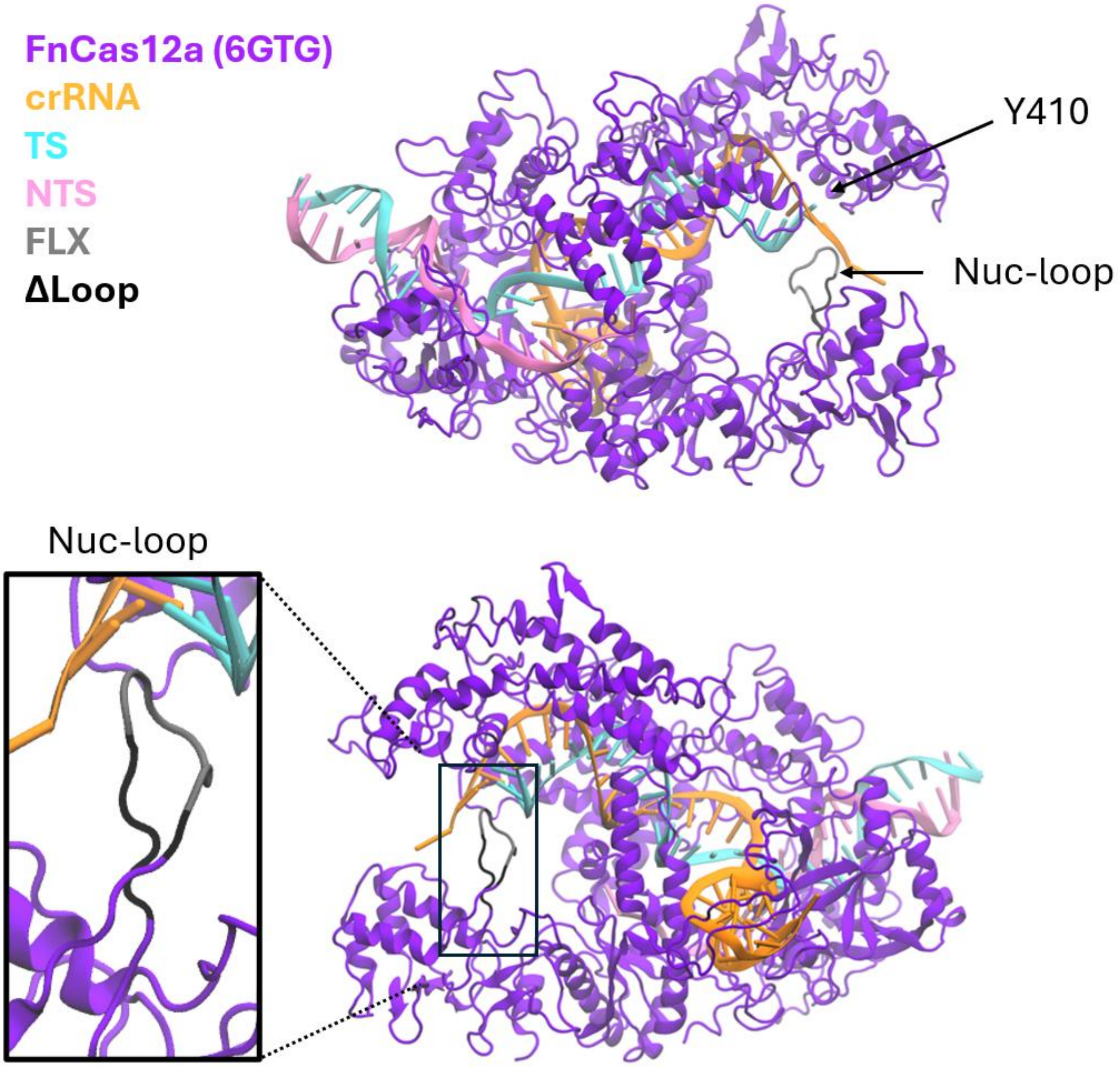

Figure S9: *Structure of FnCas12a (6GTG), showing polypeptide (purple), crRNA (orange), target-strand DNA (cyan), and non-target strand DNA (mauve). Arrows show REC2 gate (Y410) and Nuc-loop. Inset highlights amino acids in FLX substitution (grey) and ΔLoop deletion (black). Deletion is inclusive of grey region highlighted.*

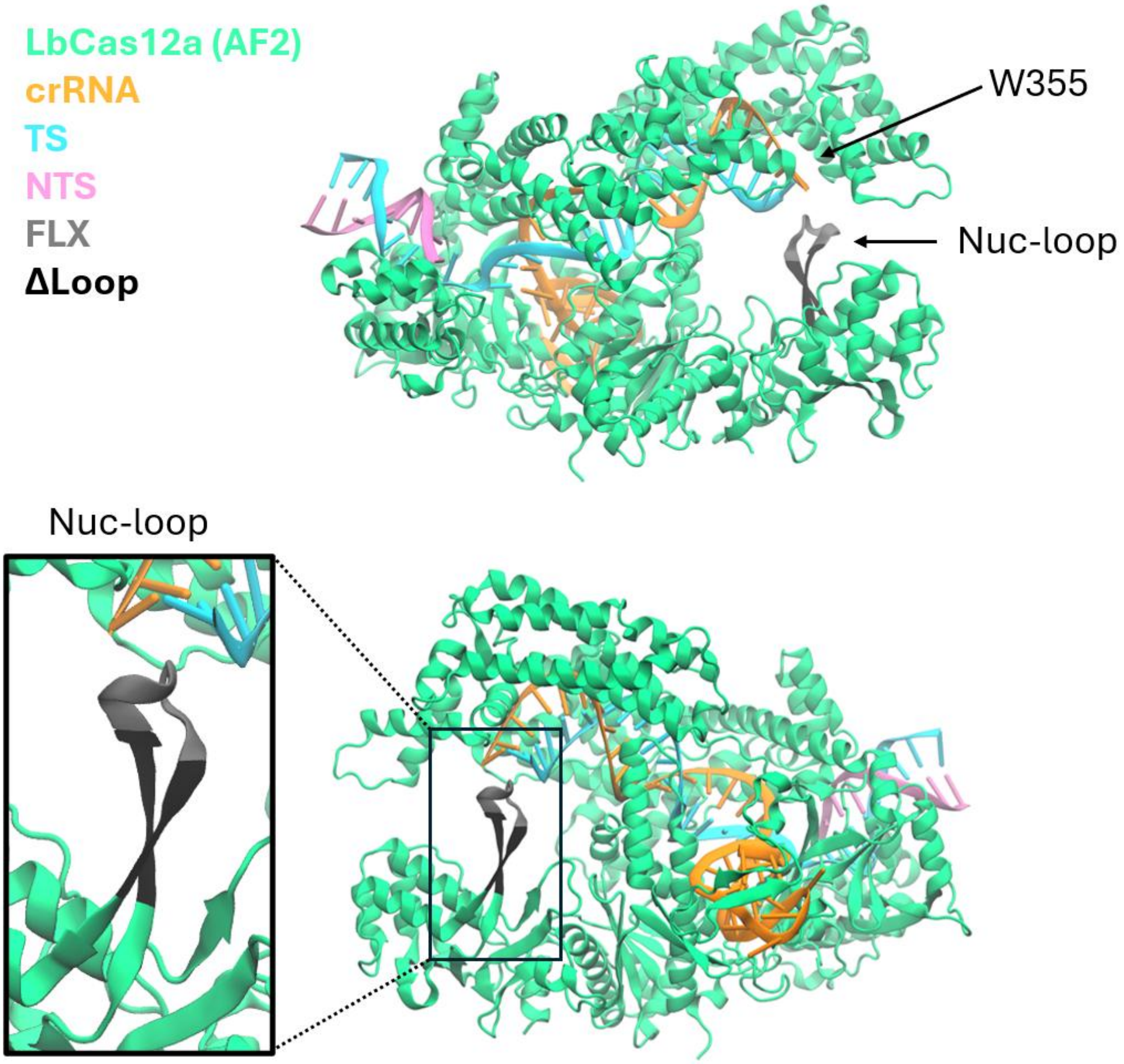

Figure S10: *Structure of LbCas12a (AF2), showing polypeptide (green), crRNA (orange), target-strand DNA (cyan), and non-target strand DNA (mauve) -nucleic acid structures from 5XUS. Arrows show REC2 gate (W355) and Nuc-loop. Inset highlights amino acids in FLX substitution (grey) and ΔLoop deletion (black). Deletion is inclusive of grey region highlighted.*

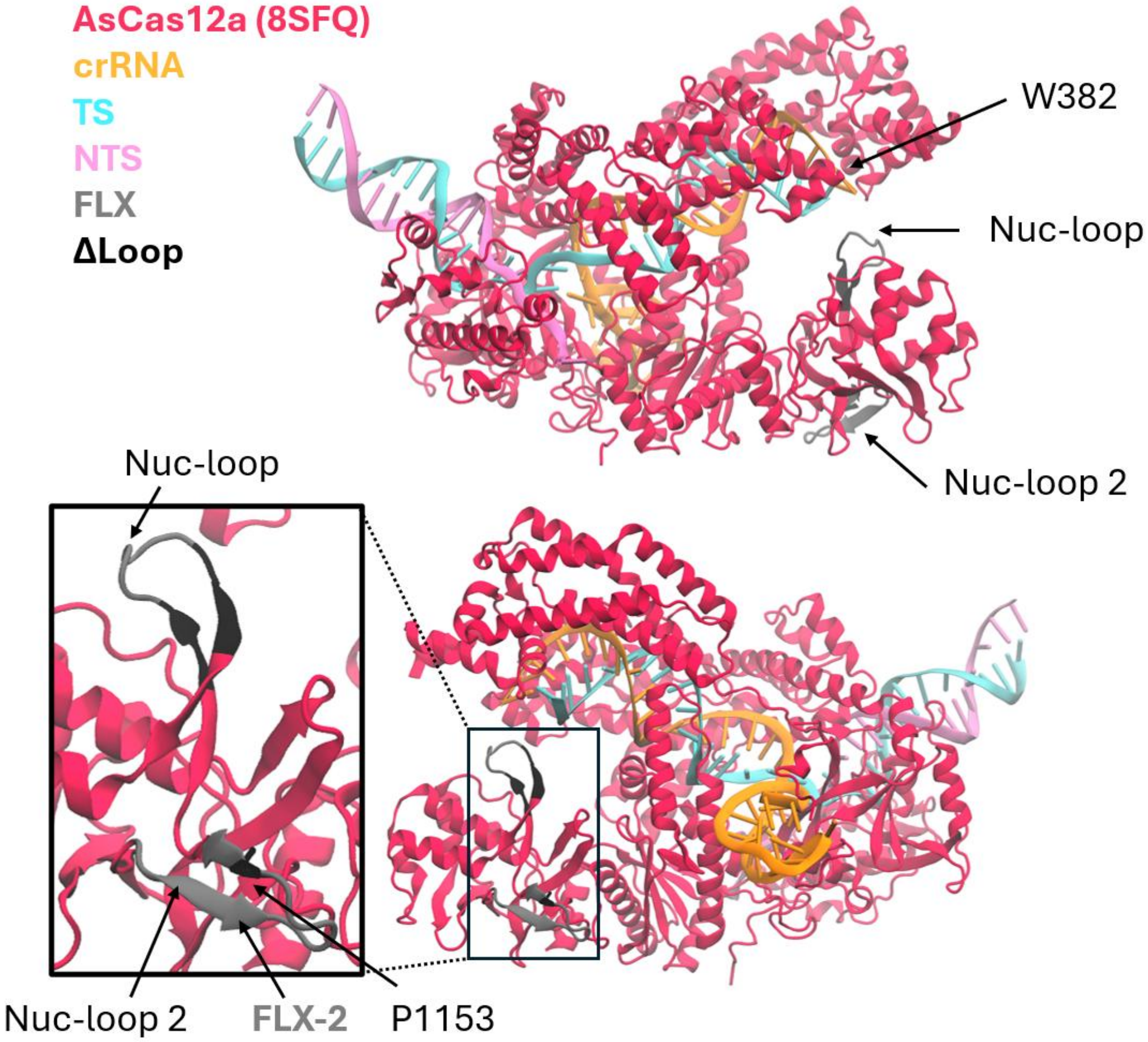

Figure S11: *Structure of AsCas12a (8SFQ), showing polypeptide (red), crRNA (orange), target-strand DNA (cyan), and non-target strand DNA (mauve). Arrows show REC2 gate (W382), P1153, Nuc-loop, and Nuc-loop 2. Inset highlights amino acids in FLX substitution (grey) and ΔLoop deletion (black). Deletion is inclusive of grey region highlighted.*

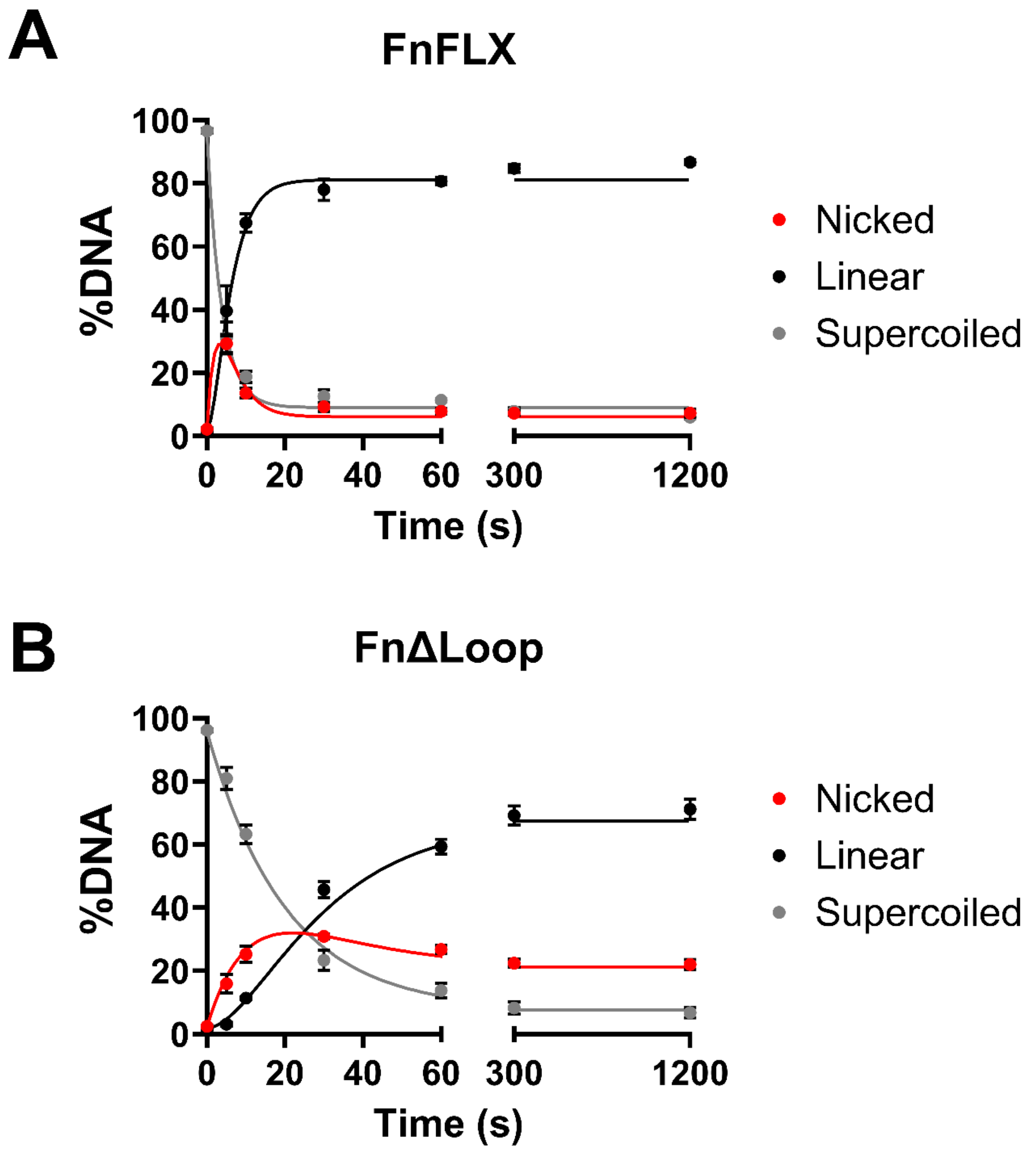

Figure S12: *Quantification of DNA fractions over time, with Cas12a mutant indicated. Line shows fit of mean rate constants, dots show mean %DNA, error bars ± s.d.*

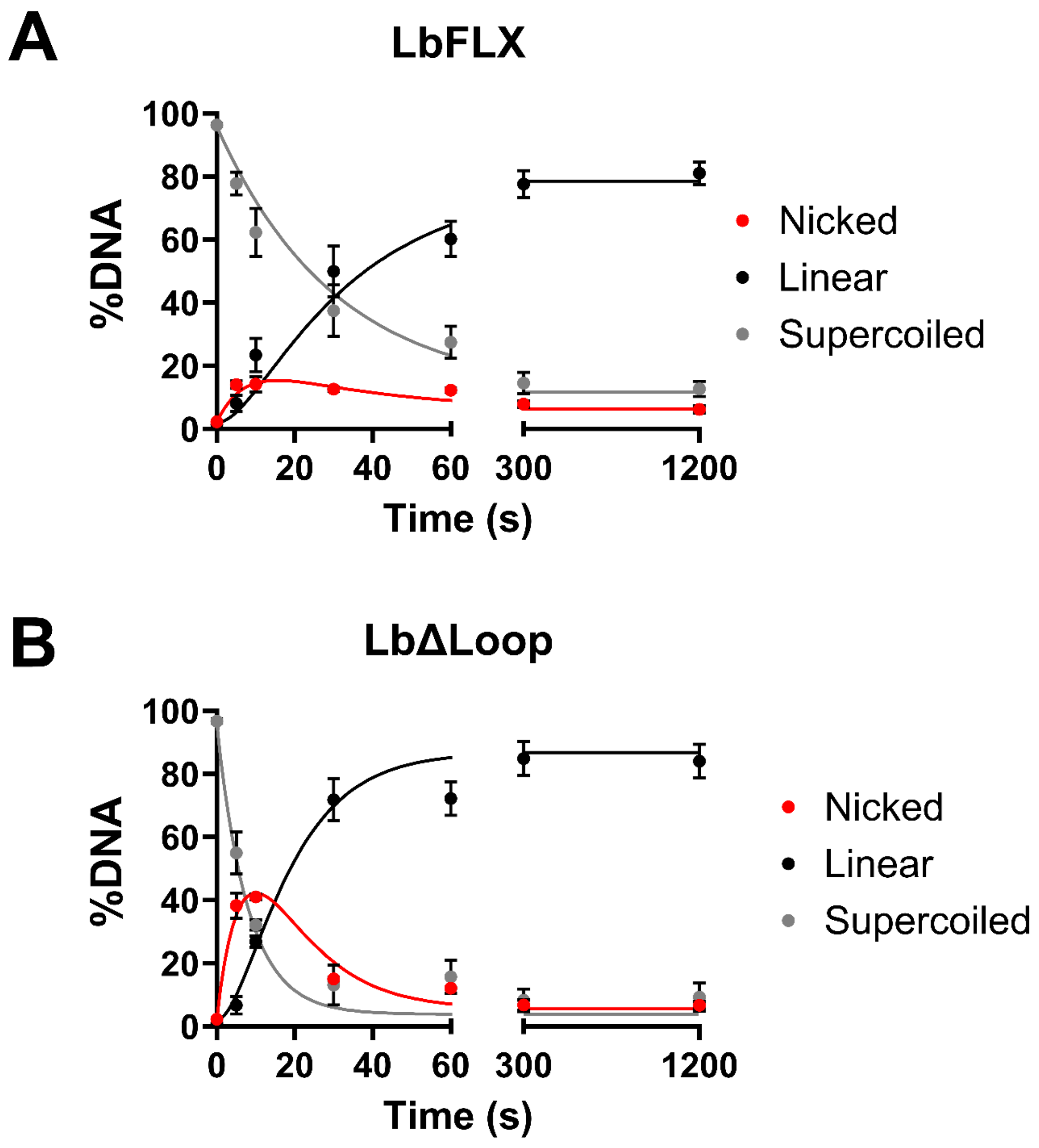

Figure S13: *Quantification of DNA fractions over time, with Cas12a mutant indicated. Line shows fit of mean rate constants, dots show mean %DNA, error bars ± s.d.*

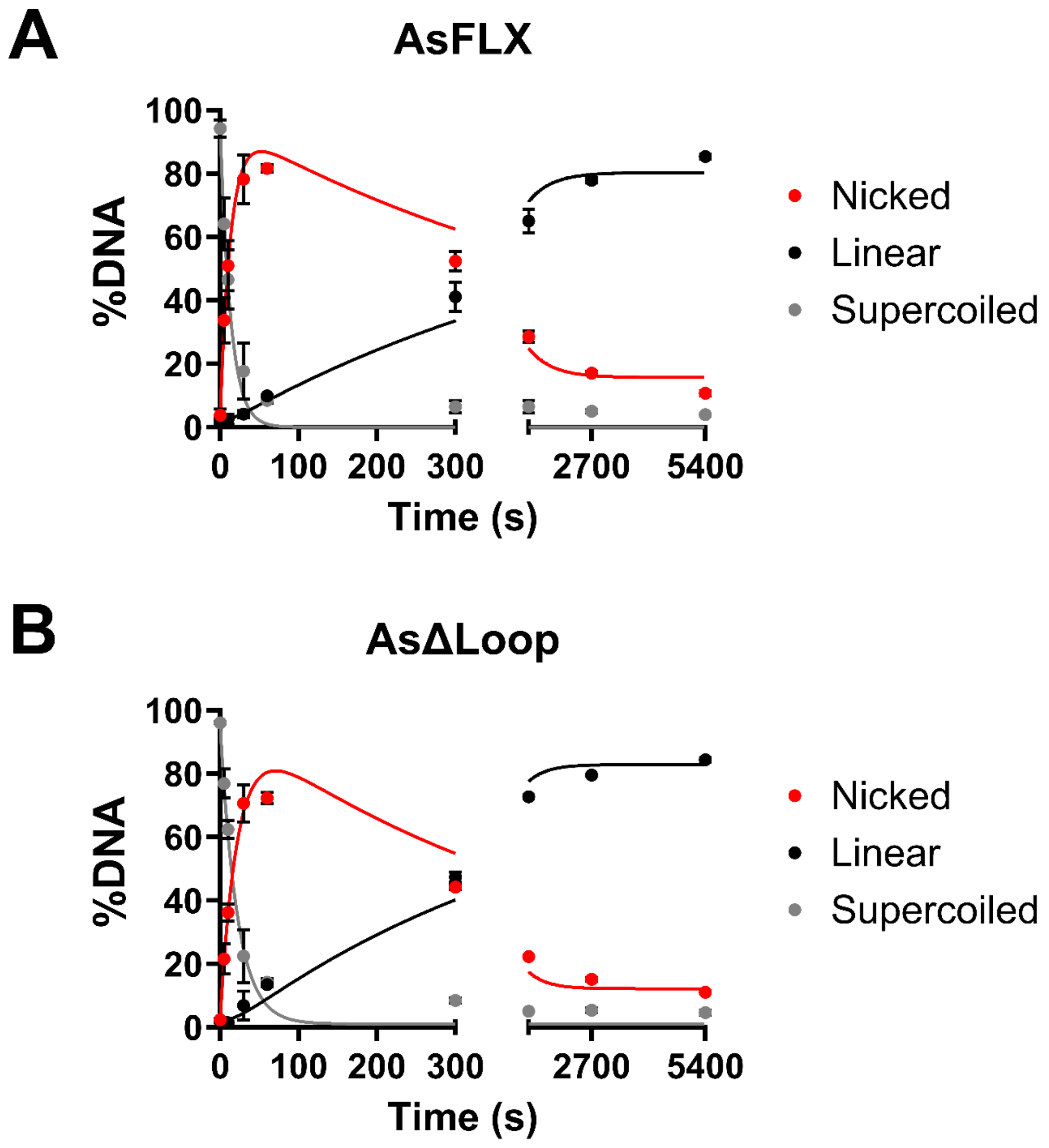

Figure S14: *Quantification of DNA fractions over time, with Cas12a mutant indicated. Line shows fit of mean rate constants, dots show mean %DNA, error bars ± s.d.*

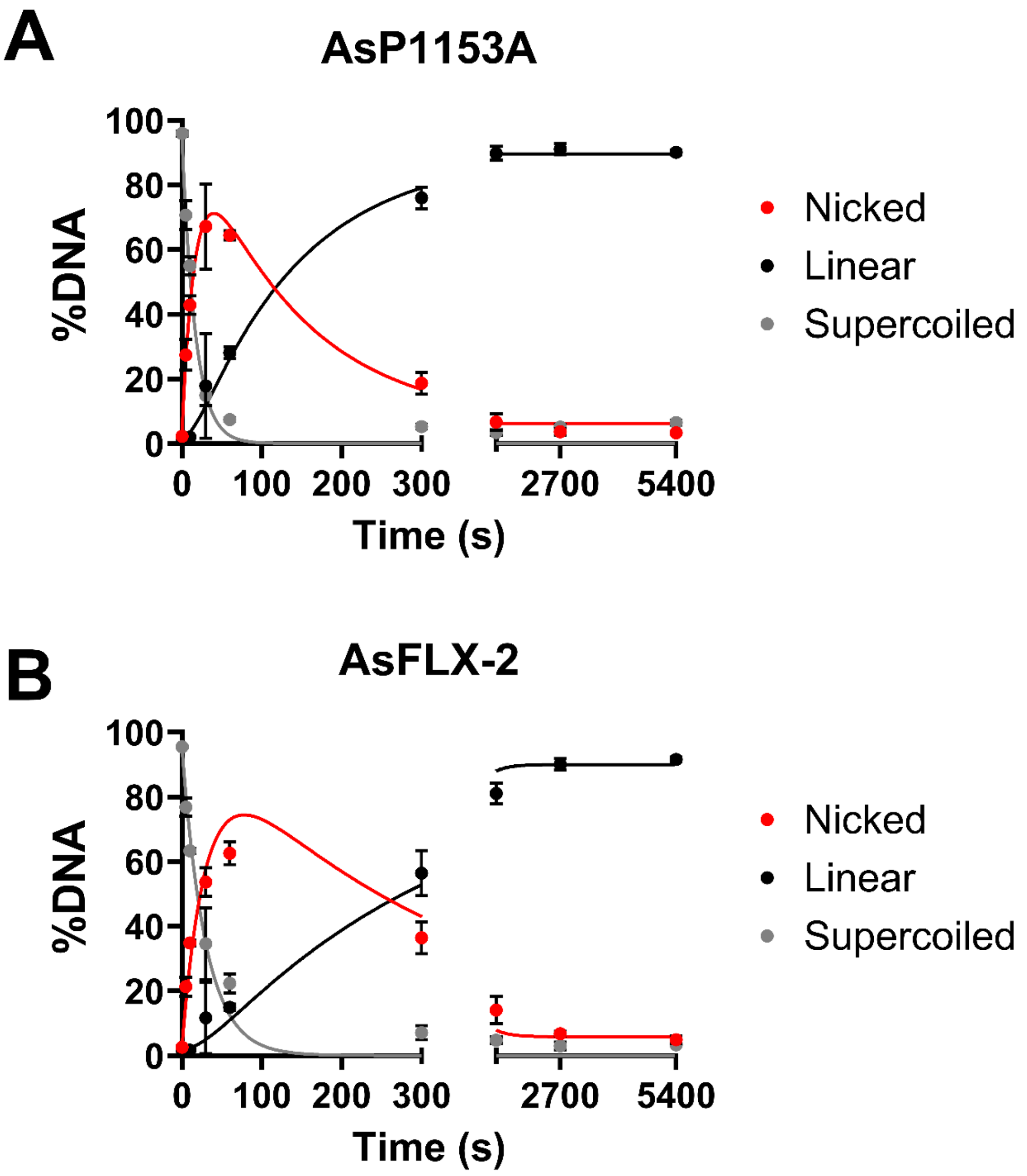

Figure S15: *Quantification of DNA fractions over time, with Cas12a mutant indicated. Line shows fit of mean rate constants, dots show mean %DNA, error bars ± s.d.*

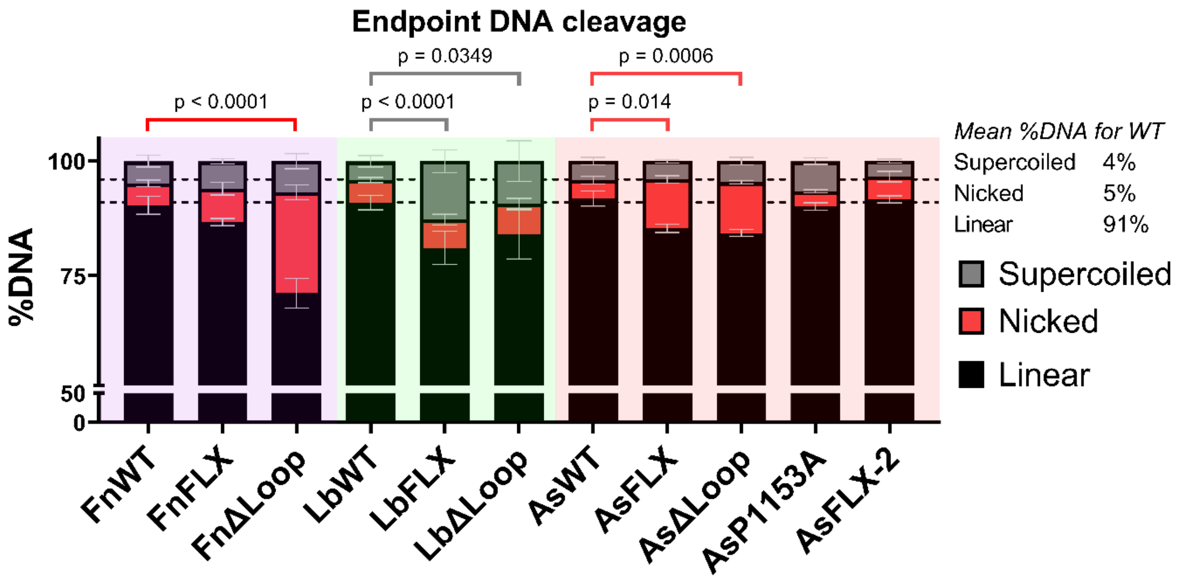

Figure S16: *Comparison of DNA fractions at endpoint of cis cleavage assays. Complete cis cleavage results in linearised plasmid DNA (black), partial cleavage leaves nicked plasmid products (red), and some fraction of target plasmid remains uncut and negatively supercoiled (grey). Bars show mean, error bars ± s.d. Dotted horizontal lines show the mean of DNA fraction across WT FnCas12a, LbCas12a, and AsCas12a. Grey brackets show statistical comparisons of supercoiled (uncut) DNA at endpoint of reaction. Red brackets show statistical comparisons of nicked plasmid DNA. Statistical significance evaluated by two-way ANOVA with Tukey's multiple comparison test.*

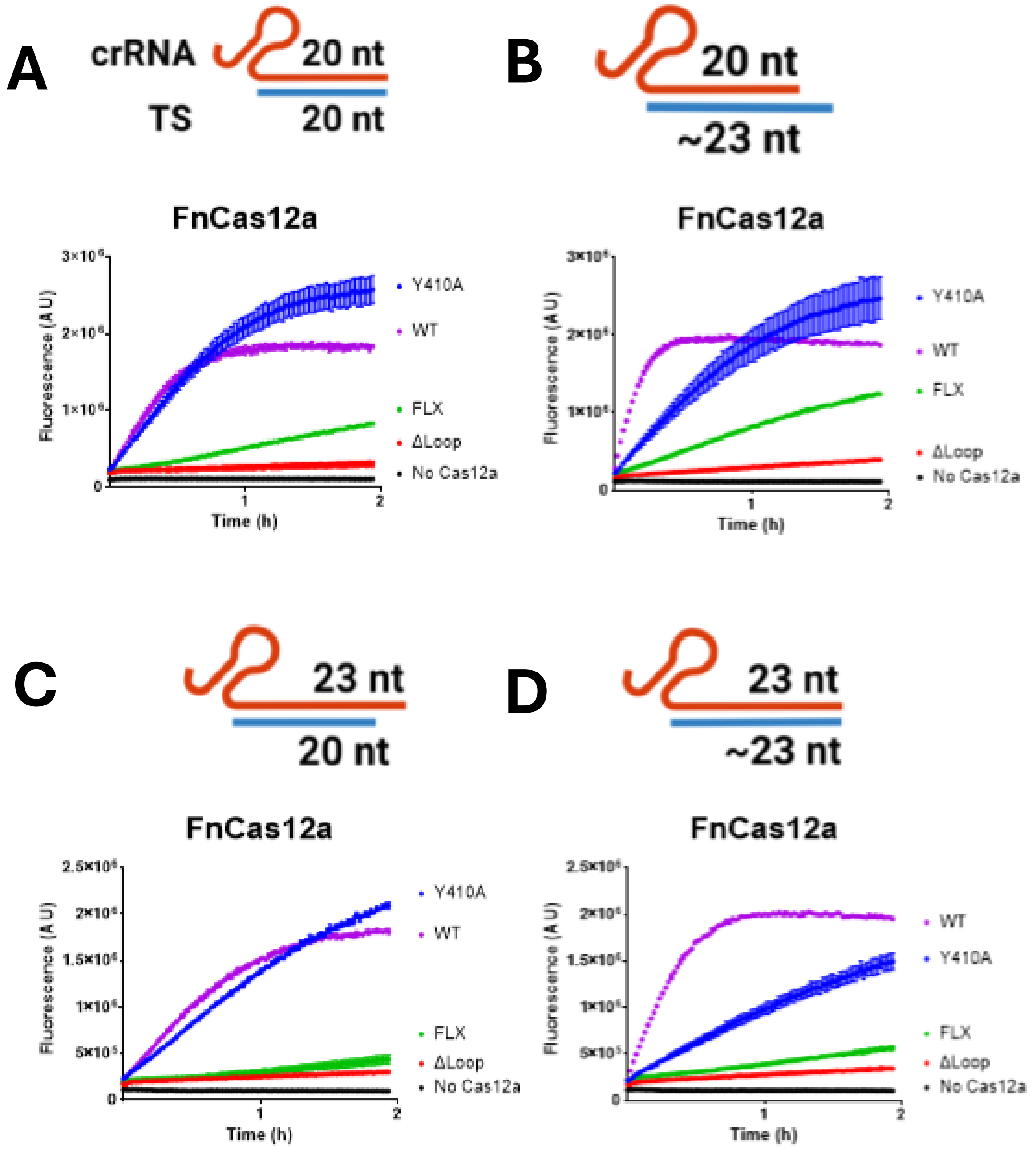

Figure S17: *Trans cleavage curves of FnCas12a WT and mutants, activated by crRNA and target-strand combinations with spacer lengths of (A) crRNA 20nt & TS 20nt, (B) crRNA 20nt & TS ~23nt, (C) crRNA 23nt & TS 20nt, and (D) crRNA 23nt and TS ~23nt. Points show mean, and error bars s.d.*

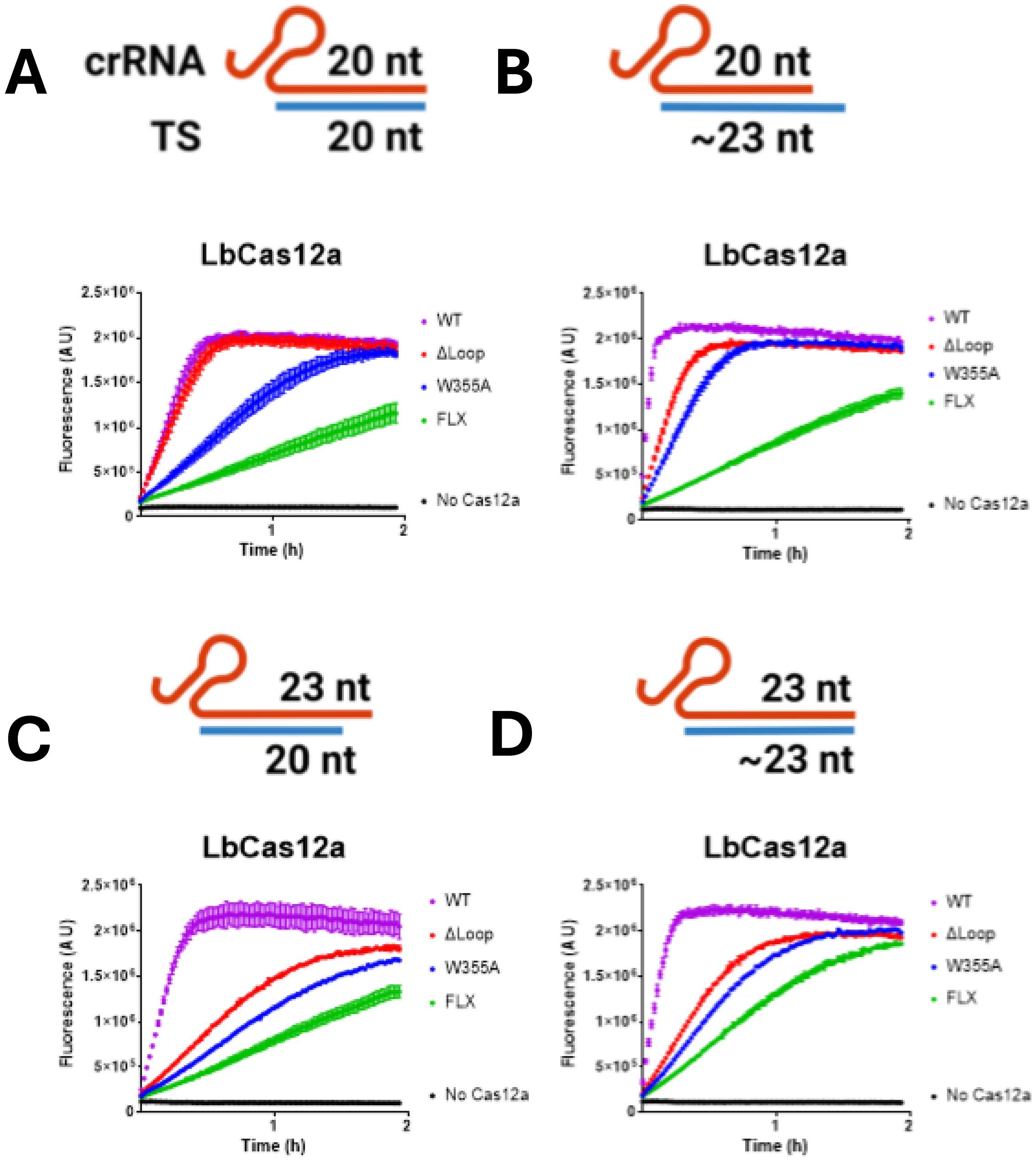

Figure S18: *Trans cleavage curves of LbCas12a WT and mutants, activated by crRNA and target-strand combinations with spacer lengths of (A) crRNA 20nt & TS 20nt, (B) crRNA 20nt & TS ~23nt, (C) crRNA 23nt & TS 20nt, and (D) crRNA 23nt and TS ~23nt. Points show mean, and error bars s.d.*

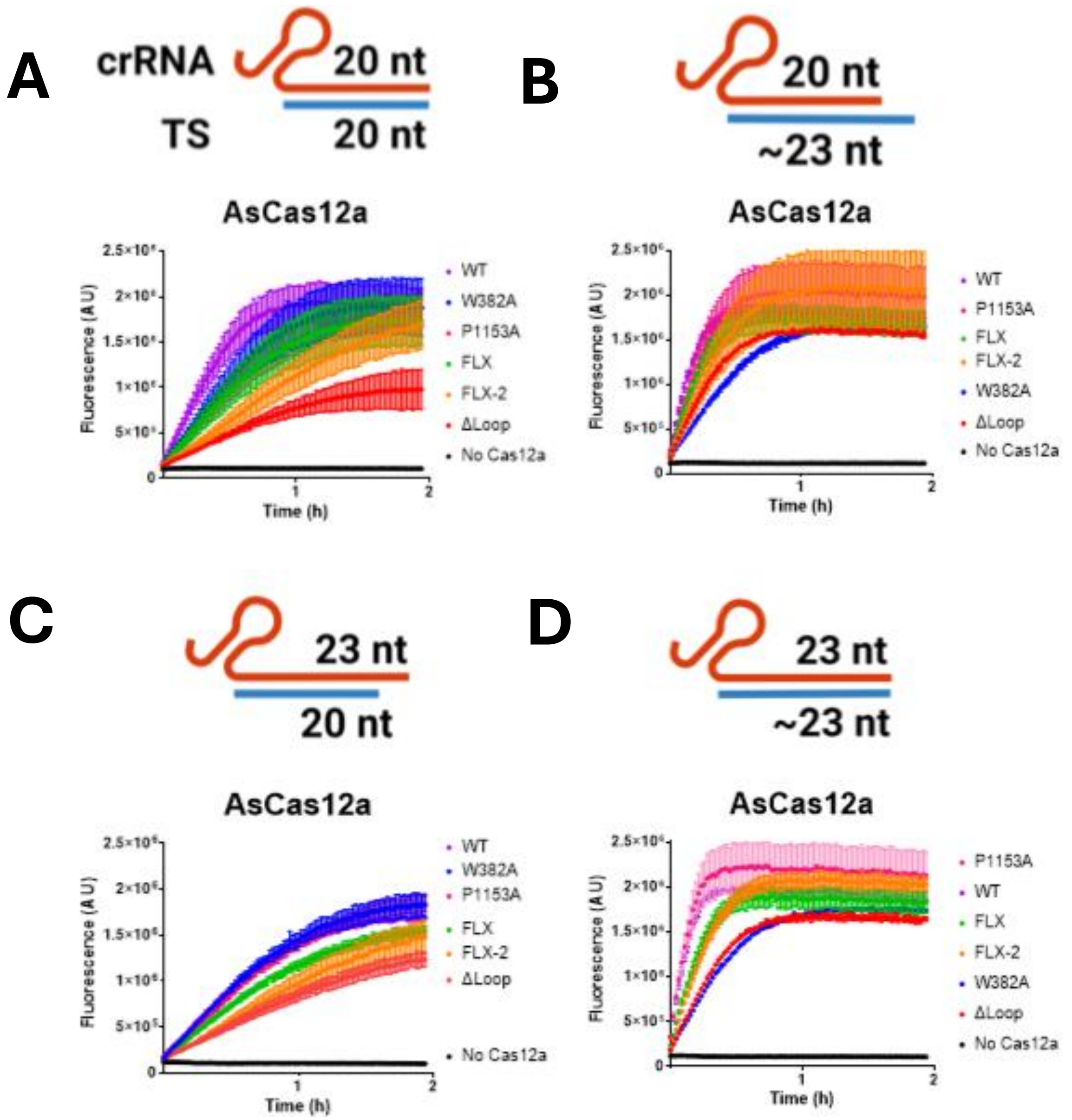

Figure S19: *Trans cleavage curves of AsCas12a WT and mutants, activated by crRNA and target-strand combinations with spacer lengths of (A) crRNA 20nt & TS 20nt, (B) crRNA 20nt & TS ~23nt, (C) crRNA 23nt & TS 20nt, and (D) crRNA 23nt and TS ~23nt. Points show mean, and error bars s.d.*

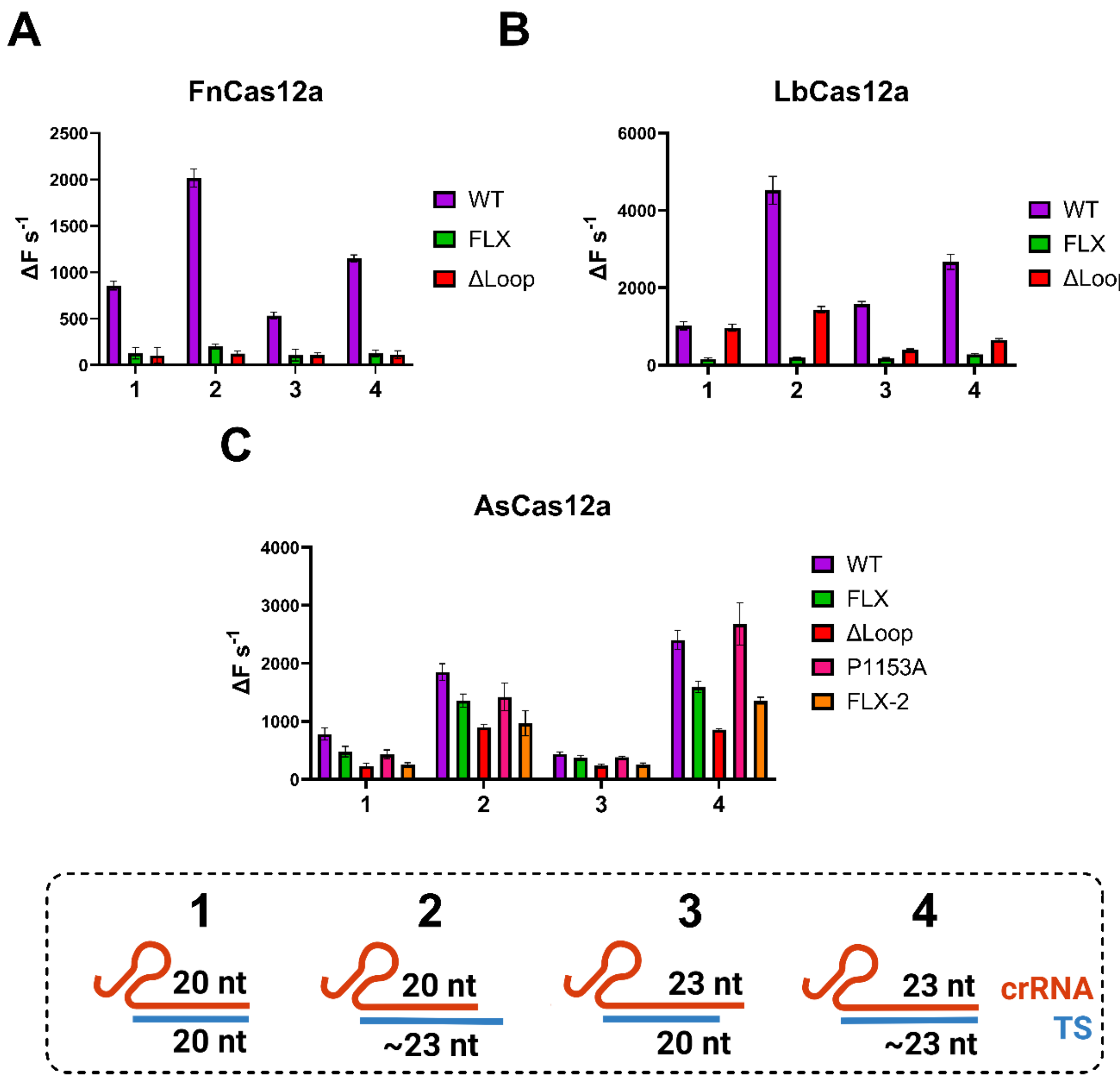

FigureS20: *Quantification of slope of first 300s of trans cleavage curves, as determined by linear regression of each replicate. Bar shows mean, error bars show 95% confidence intervals. Calculated in Graphpad Prism 10.*

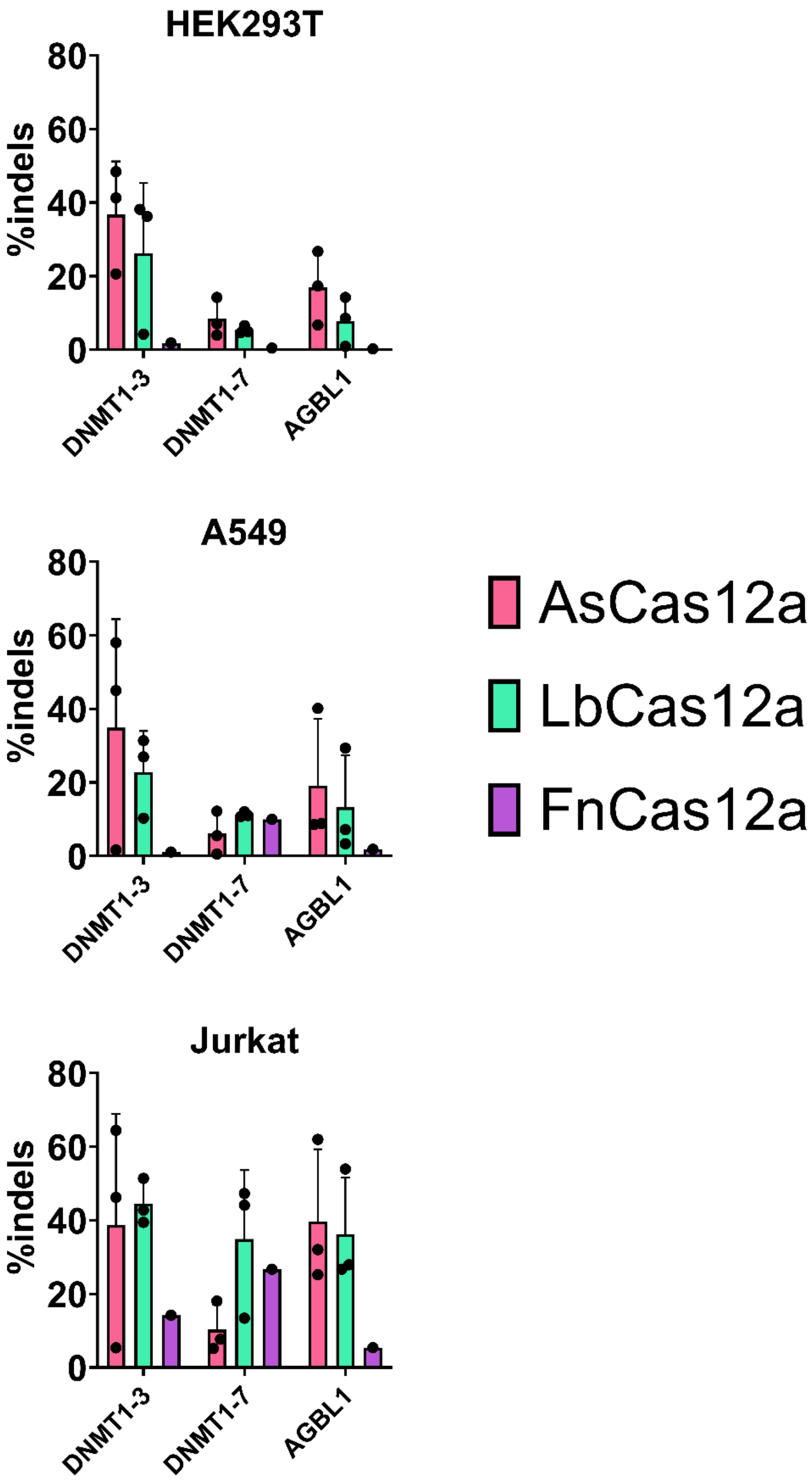

Figure S21: *Gene editing with crRNA as indicated. Performed in (A) HEK293T cells, (B) A549 cells, and (C) Jurkat cells. Dots shown individual values, bar shows mean, error bars s.d. Note, n = 1 transformations performed for FnCas12a.*

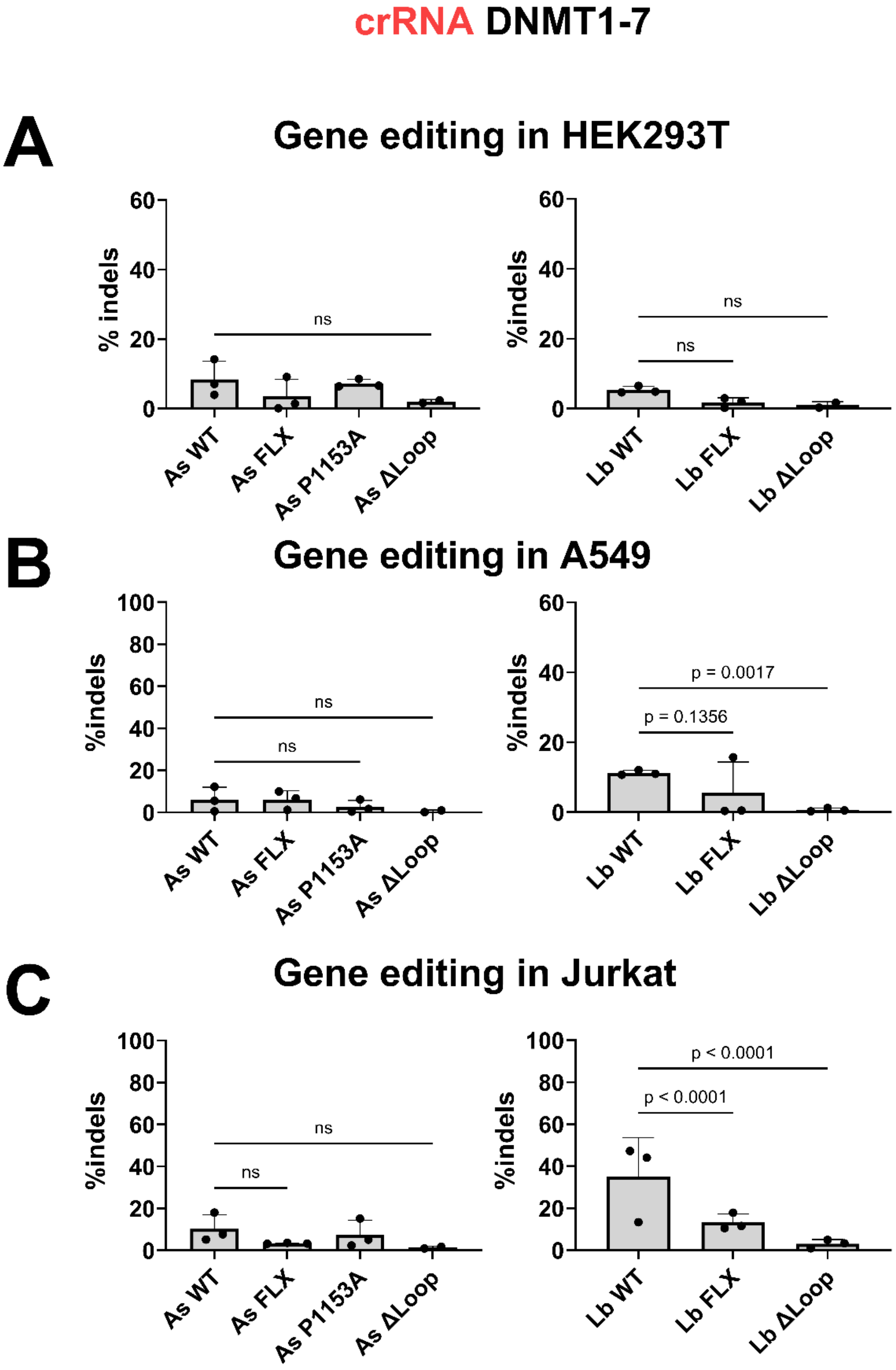

Figure S22: *Gene editing with crRNA DNMT1-7. Performed in (A) HEK293T cells, (B) A549 cells, and (C) Jurkat cells. Bars show mean, error bars s.d. Statistical significance evaluated by two-way ANOVA with Tukey's multiple comparison test.*

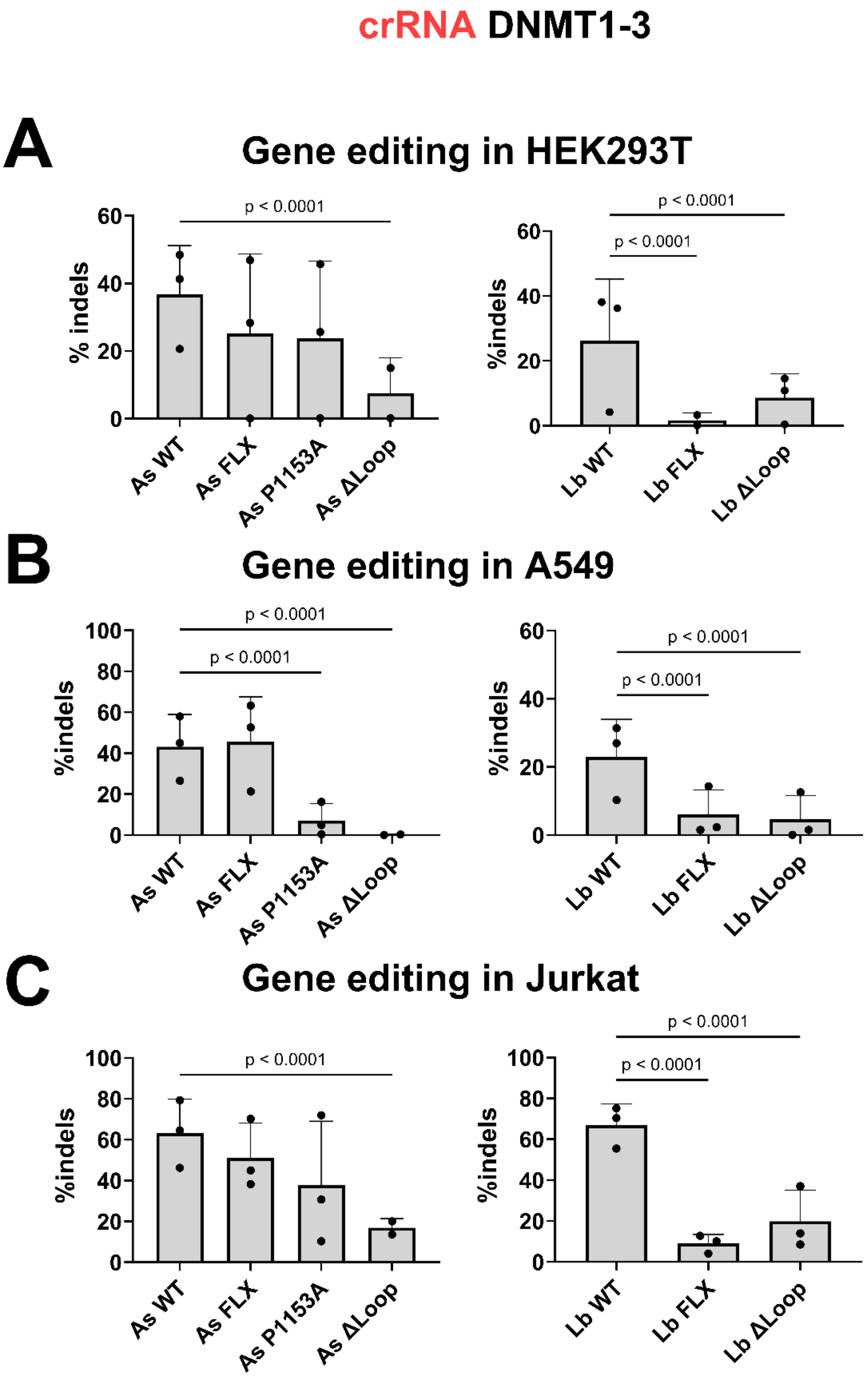

Figure S23: *Gene editing with crRNA DNMT1-3. Performed in (A) HEK293T cells, (B) A549 cells, and (C) Jurkat cells. Bars show mean, error bars s.d. Statistical significance evaluated by two-way ANOVA with Tukey's multiple comparison test .*

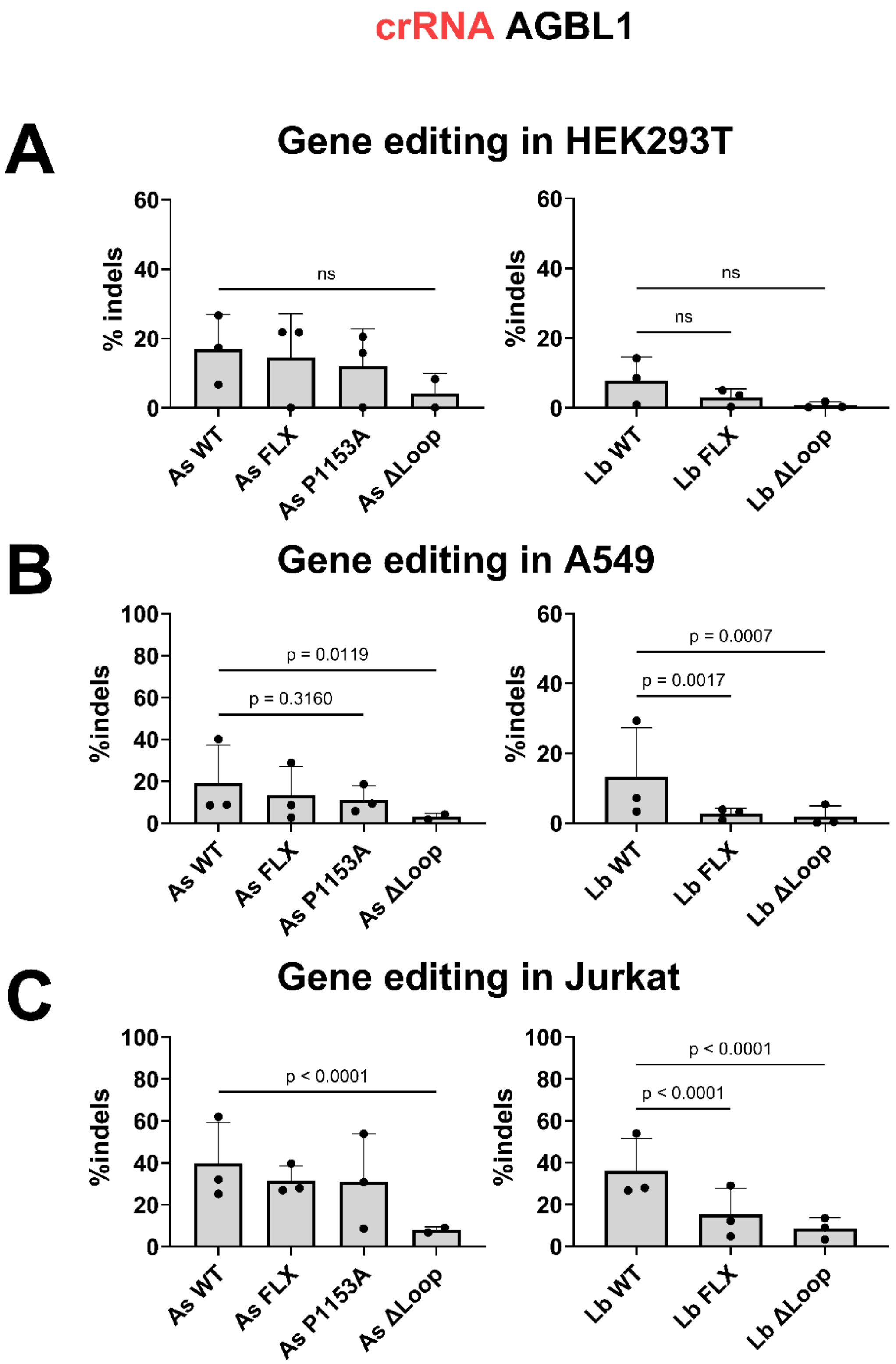

Figure S24: *Gene editing with crRNA AGBL1. Performed in (A) HEK293T cells, (B) A549 cells, and (C) Jurkat cells. Bars show mean, error bars s.d. Statistical significance evaluated by two-way ANOVA with Tukey's multiple comparison test.*

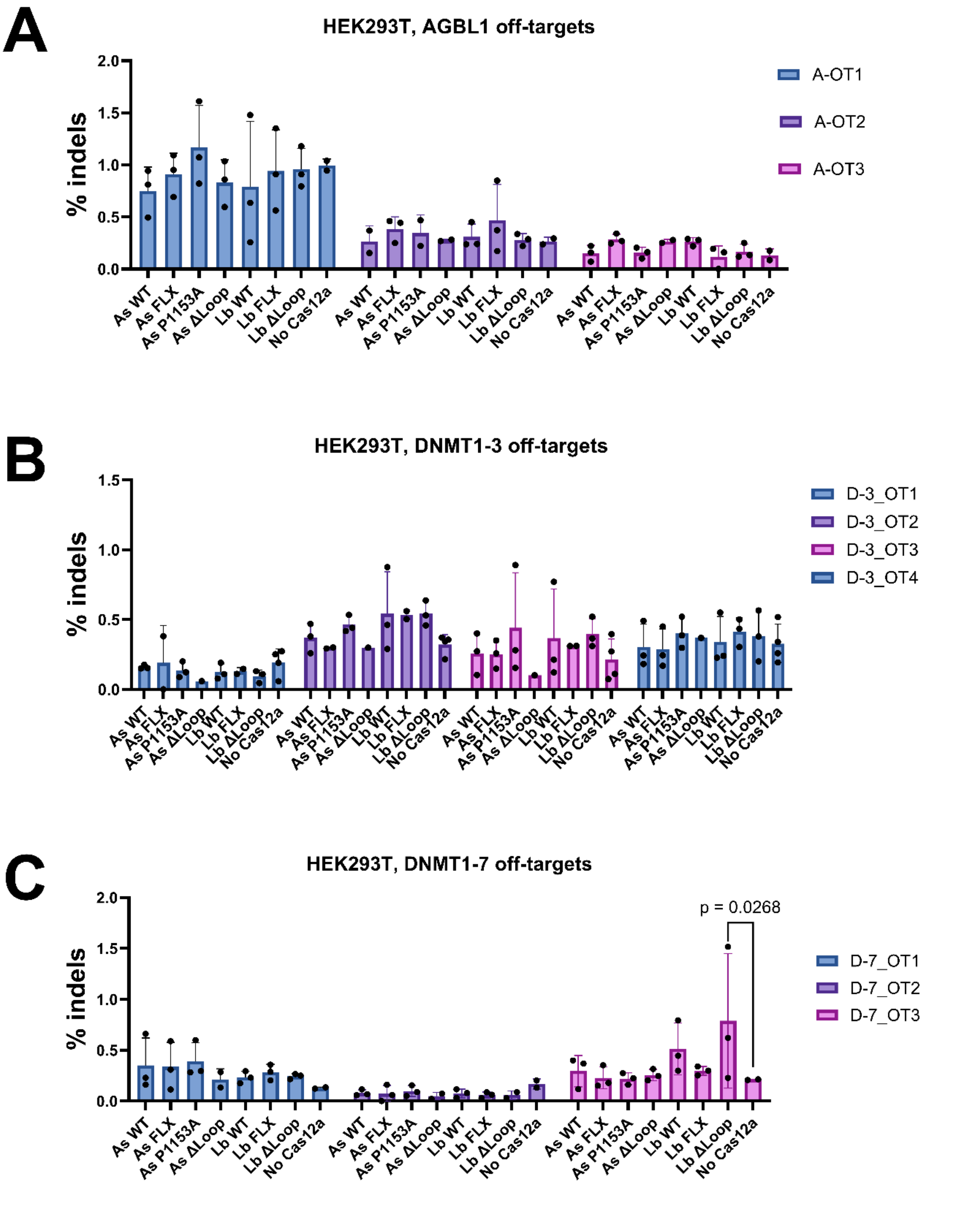

Figure S25: *Off-target (OT) editing in HEK293T cell line. Highest represented off-target sites for (A) AGBL1, (B) DNMT1-3, and (C) DNMT1-7, as identified in Kleinstiver 2019 (https://doi.org/10.1038/s41587-018-0011-0). Bars shown mean, error bars s.d. Statistical significance evaluated by two-way ANOVA with Tukey's multiple comparison test.*

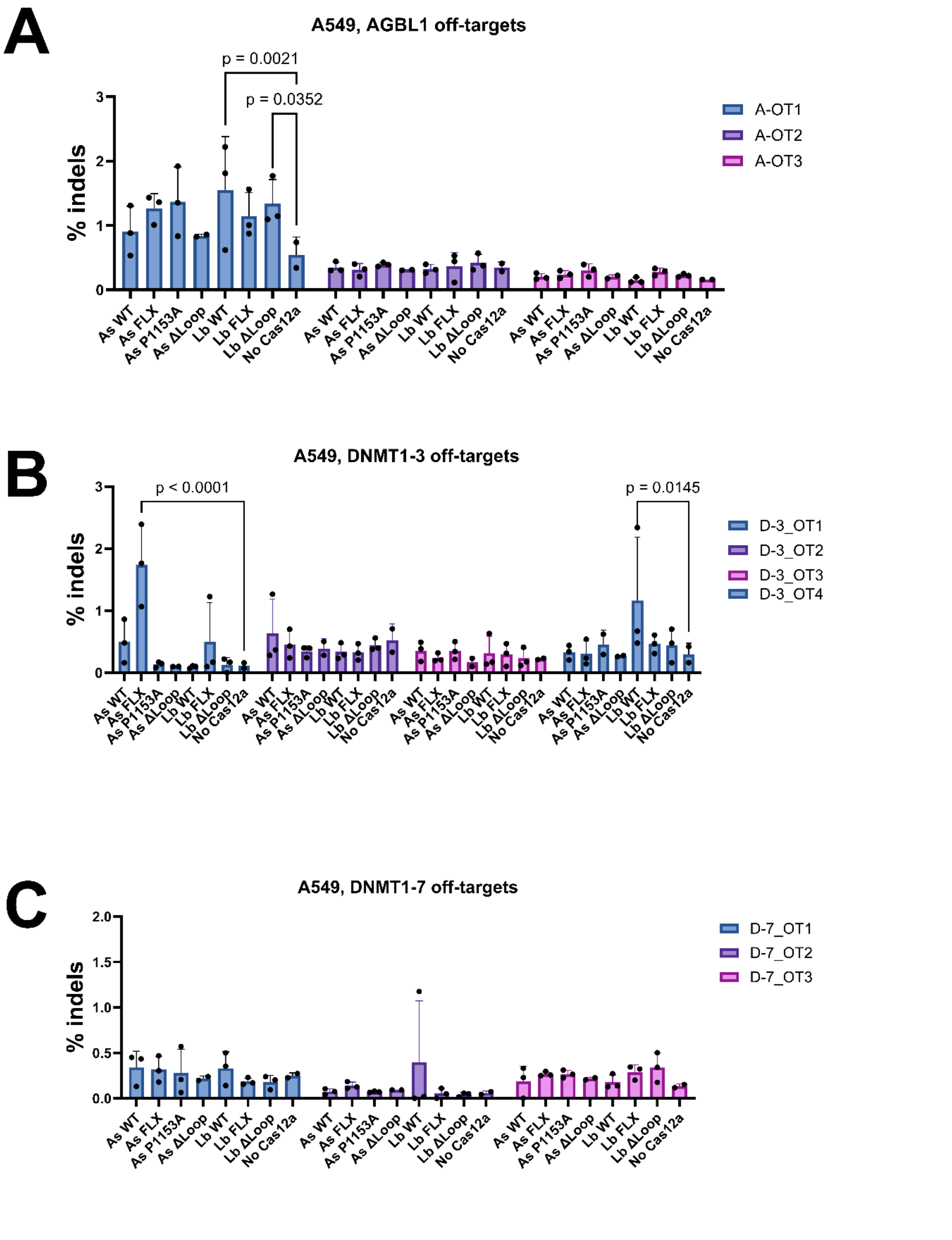

Figure S26: *Off-target (OT) editing in A549 cell line. Highest represented off-target sites for (A) AGBL1, (B) DNMT1-3, and (C) DNMT1-7, as identified in Kleinstiver 2019 (https://doi.org/10.1038/s41587-018-0011-0). Bars shown mean, error bars s.d. Statistical significance evaluated by two-way ANOVA with Tukey's multiple comparison test.*

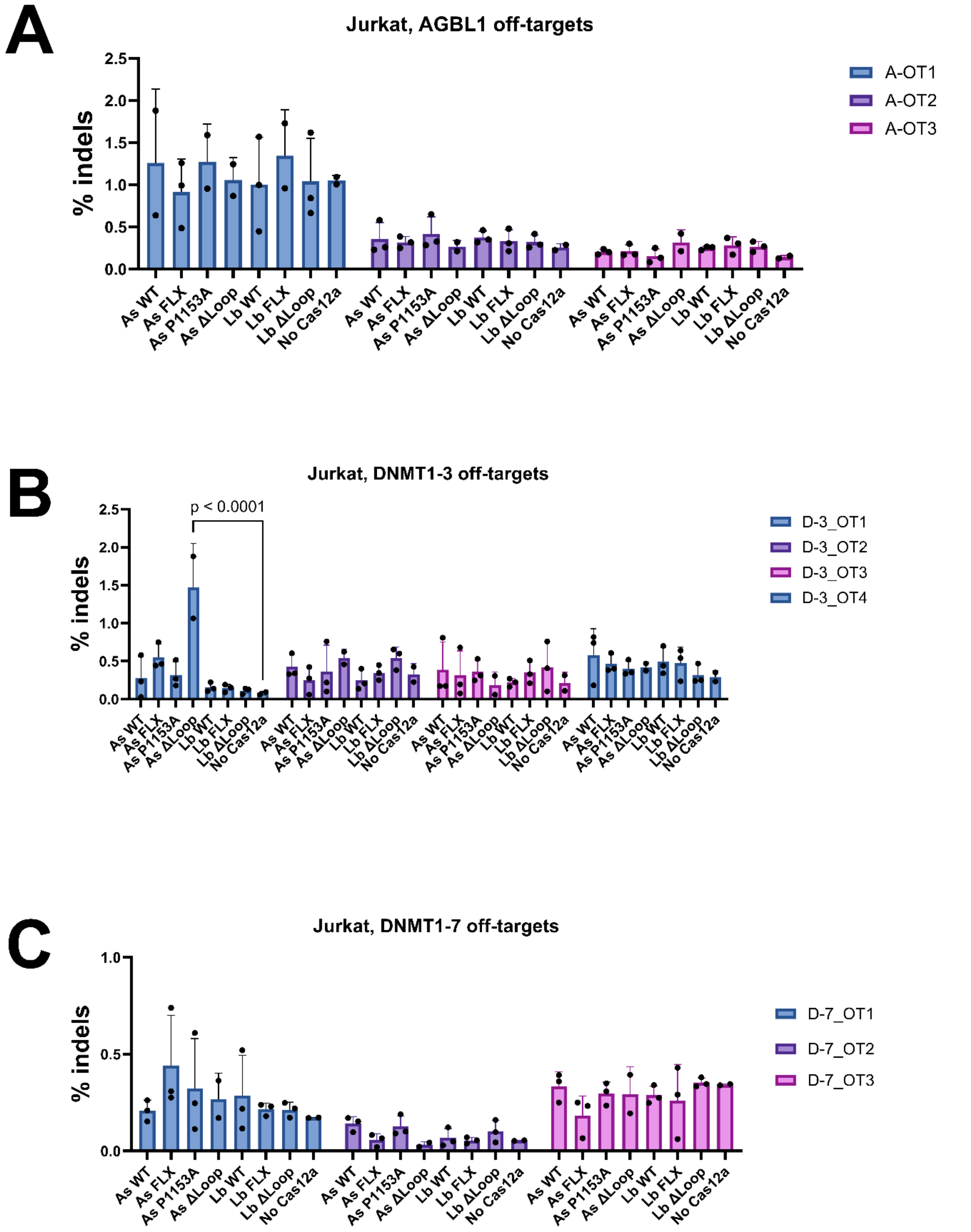

Figure S27: *Off-target (OT) editing in Jurkat cell line. Highest represented off-target sites for (A) AGBL1, (B) DNMT1-3, and (C) DNMT1-7, as identified in Kleinstiver 2019 (https://doi.org/10.1038/s41587-018-0011-0). Bars shown mean, error bars s.d. Statistical significance evaluated by two-way ANOVA with Tukey's multiple comparison test.*

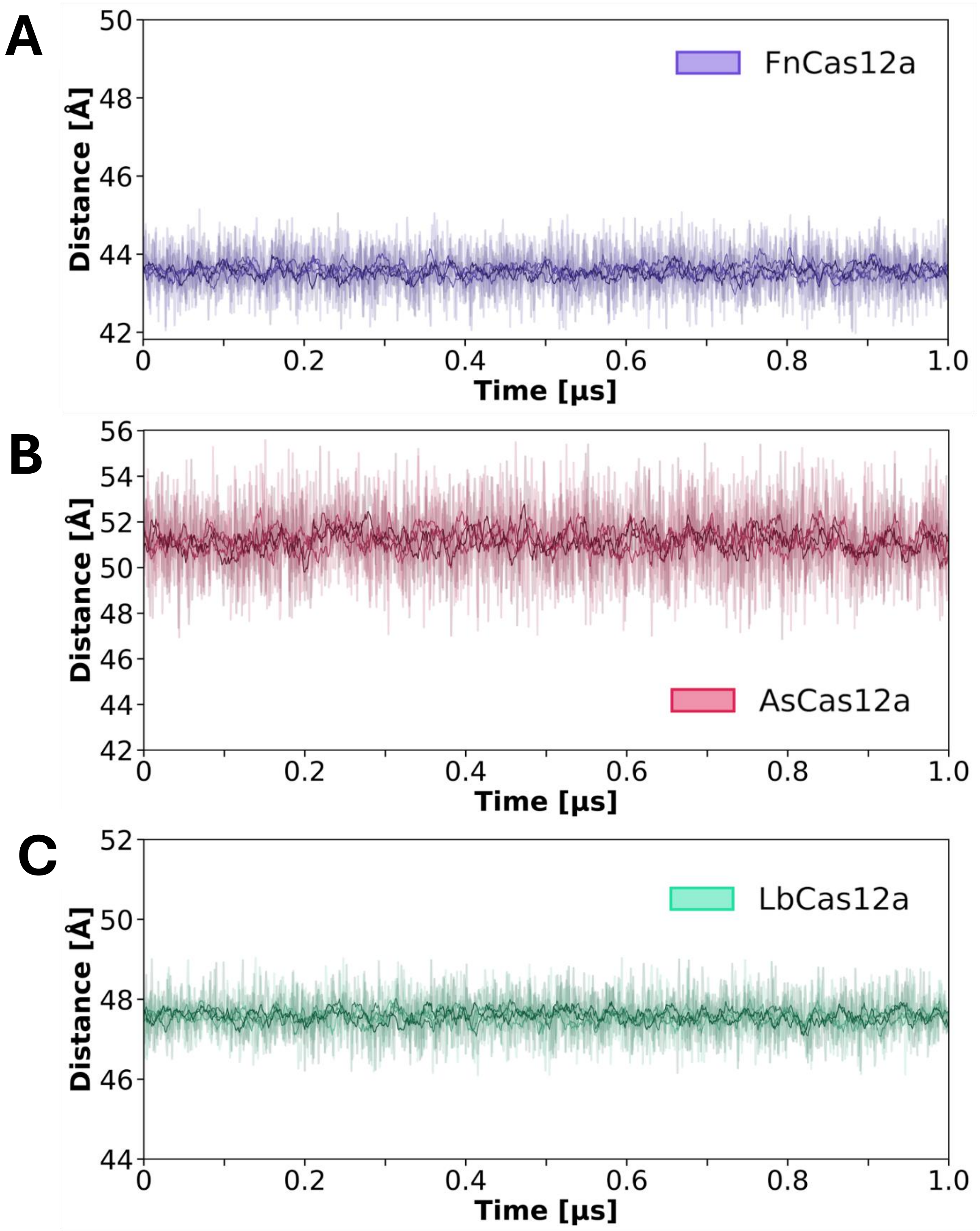

Figure S28: *Distance analysis of replicates of μs – length simulations of (A) FnCas12a, (B) AsCas12a, and (C) LbCas12a.*

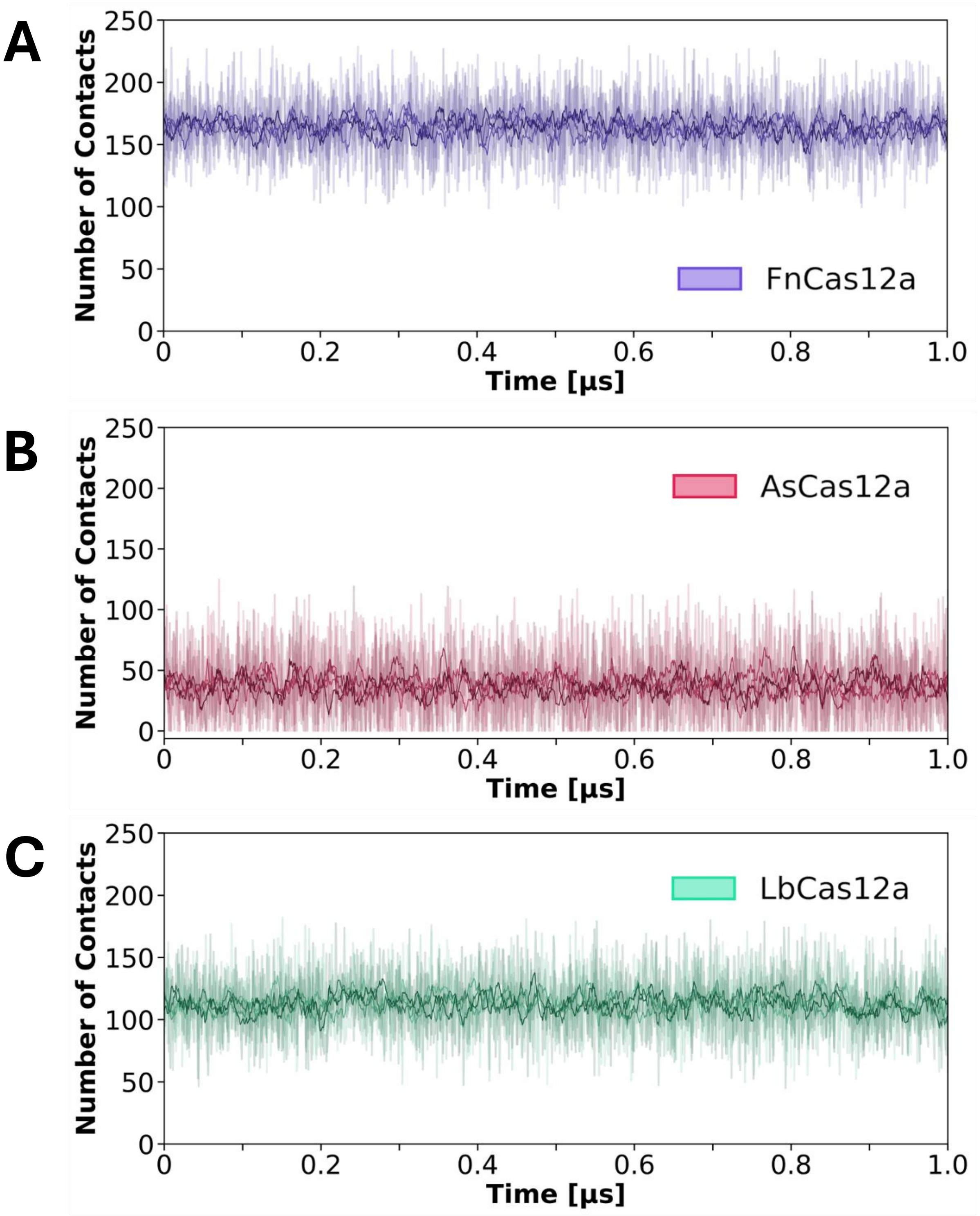

Figure S29: *Contact analysis of replicates of μs – length simulations of (A) FnCas12a, (B) AsCas12a, and (C) LbCas12a.*

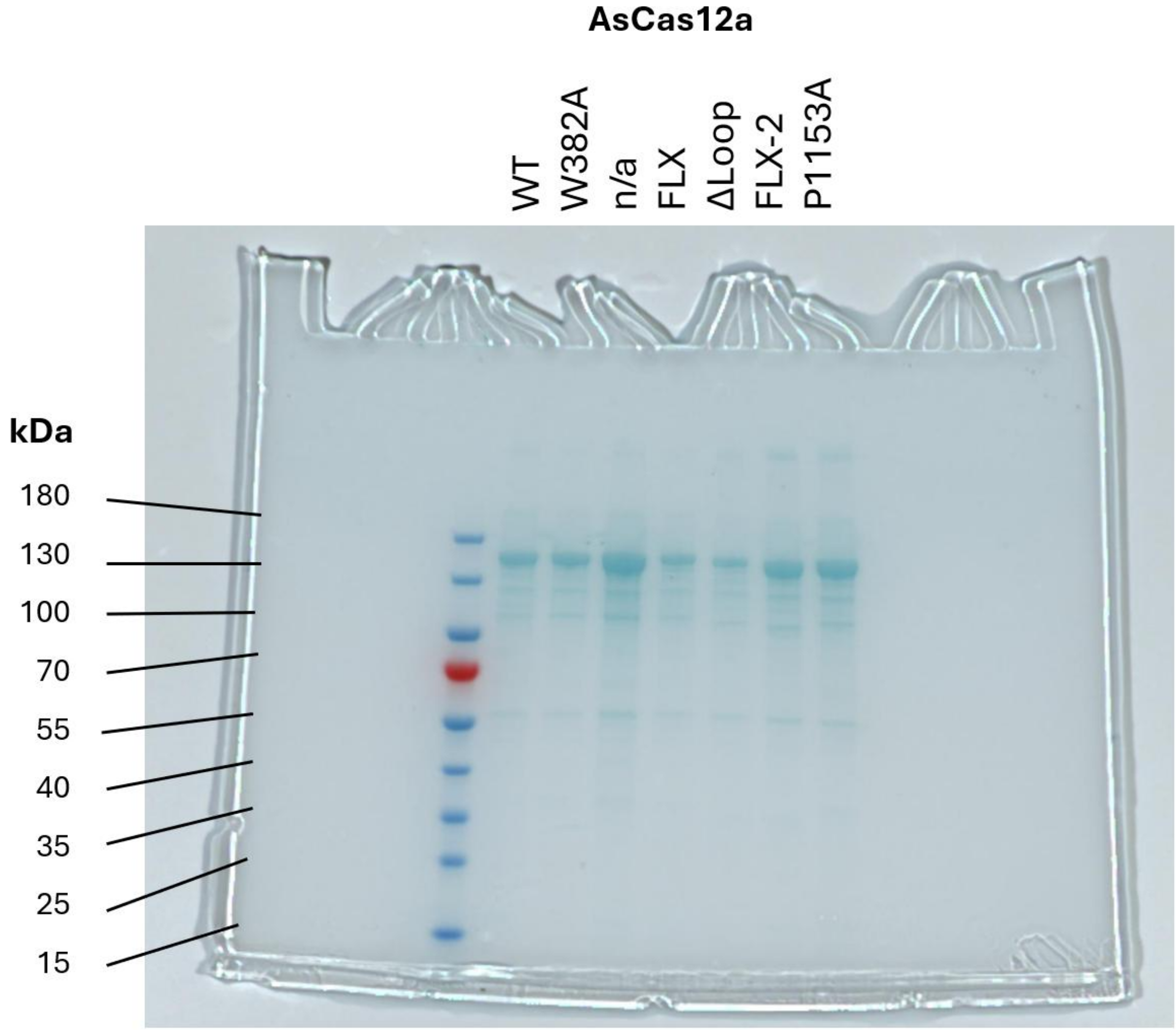

Figure S30: *SDS-PAGE of AsCas12a nucleases. N/A indicates nuclease not used herein.*

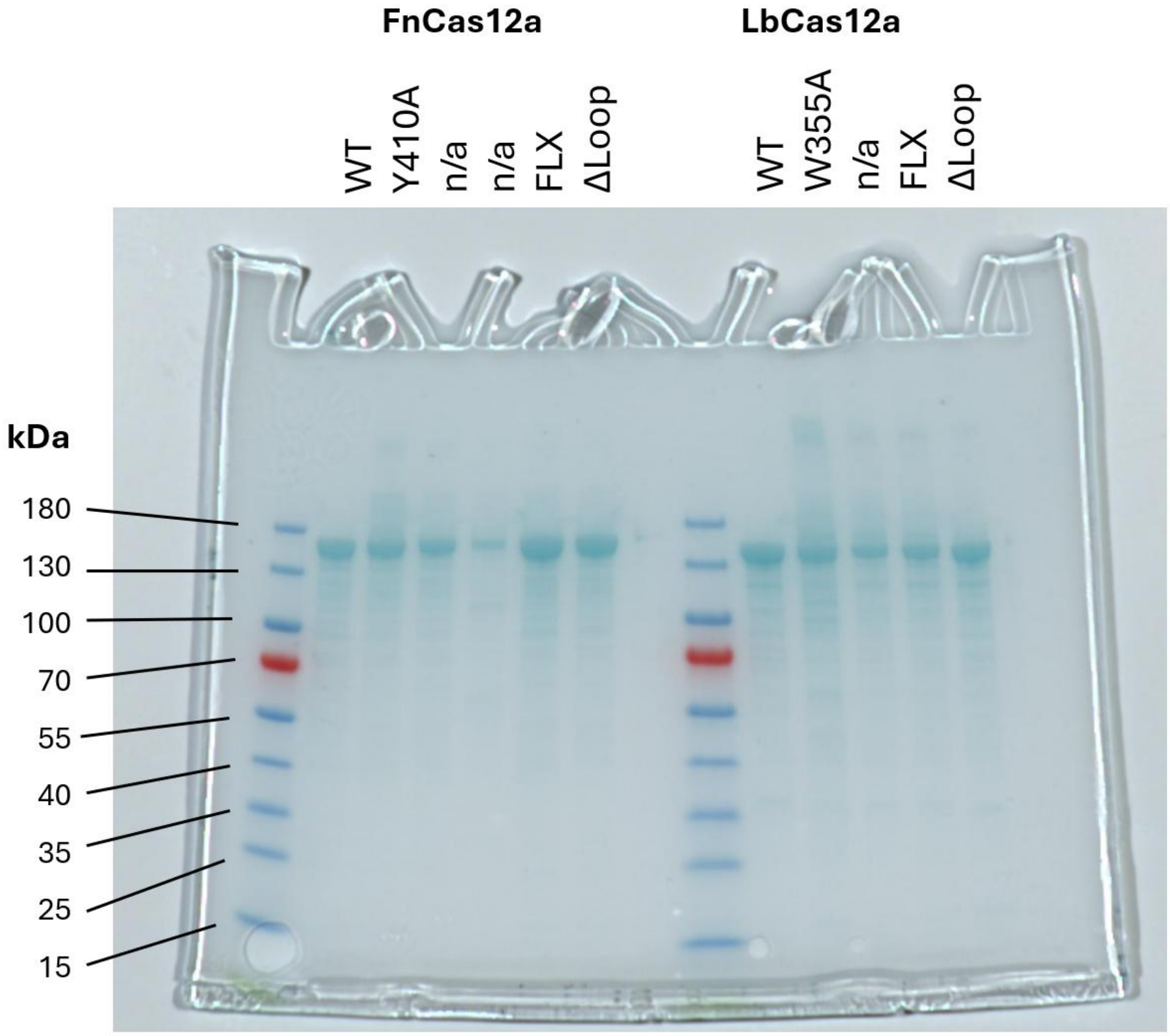

Figure S31: *SDS-PAGE of FnCas12a and LbCas12a nucleases. N/A indicates nuclease not used herein.*

Supplementary Tables

Cas12a	T (°C)	k_{NTS} (s^{-1})	k_{TS} (s^{-1})	DNA topology	Buffer
Lb[27]	25	0.203 ± 0.048	0.012 ± 0.004	(-) SC plasmid	10 mM Tris-HCl, pH 7.5, 100 mM NaCl, 10 mM $MgCl_2$, 0.1 mM DTT, 5 µg/ml BSA
Lb[26]	25	0.324 ± 0.0073	0.014 ± 0013	(-) SC plasmid	10 mM Tris-HCl, pH 7.5, 100 mM NaCl, 10 mM $MgCl_2$, 0.1 mM DTT, 5 µg/ml BSA
Lb*	30	0.112 ± 0.005	0.055 ± 0.007	(-) SC plasmid	10 mM Tris-HCl, pH 7.5, 50 mM NaCl, 10 mM $MgCl_2$, 5 µg/ml BSA, 0.1 mM DTT
As[20]	25	0.052 ± 0.6	0.0051 ± 0.2	Short linear	50 mM Na-MOPS, pH 7.0, 120 mM NaCl, 5 mM $MgCl_2$, 2 mM DTT
As*	30	0.084 ± 0.017	0.007 ± 0.002	(-) SC plasmid	10 mM Tris-HCl, pH 7.5, 50 mM NaCl, 10 mM $MgCl_2$, 5 µg/ml BSA, 0.1 mM DTT
As[25]	37	0.232 ± 0.059	0.061 ±0.010	Short linear	10 mM Tris-Cl, pH 7.9, 150 mM KCl, 5 mM $MgCl_2$, 1 mM TCEP
As[28]	37	0.029 ± 0.002	0.11 ± 0.023	Short linear	10 mM Tris-HCl, pH 8.0, 100 mM NaCl, 0.9 mM EDTA, 1 mM DTT, 5mM $MgCl_2$
As[28]	37	0.041 ± 0.012	0.19 ± 0.011	Short linear	10 mM Tris-HCl, pH 8.0, 100 mM NaCl, 0.9 mM EDTA, 1 mM DTT, 10mM $MgCl_2$
As[18]	37	0.10 ± 0.012	0.013 ± 0.0012	Short linear	50 mM HEPES, pH 7, 150 mM NaCl, 5 mM $MgCl_2$, 2 mM DTT, 0.2 mg/mL BSA
Fn*	30	0.432 ± 0.062	0.267 ± 0.038	(-) SC plasmid	10 mM Tris-HCl, pH 7.5, 50 mM NaCl, 10 mM $MgCl_2$, 5 µg/ml BSA, 0.1 mM DTT
Fn[14]	37	0.0075 ± 0.0012	0.0011 ± 0.00018	Short linear	20 mM Bicine-HCl pH 8, 150 mM KCl, 0.5mM TCEP, 5 mM $MgCl_2$
Fn[23]	37	0.0483 ± 0.006	0.0338 ± 0.002	Short linear	20 mM HEPES, pH 7.5, 150 mM KCl, 5% glycerol, and 0.5 mM DTT, 5 mM $MgCl_2$

Table S1: *Mean rates of NTS and TS cleavage (with s.d.) from published literature. *Data from this study.*

	k_{NTS} (s^{-1})			k_{TS} (s^{-1})		
	Mean	S.D.	N	Mean	S.D.	N
FnWT	0.432	0.062	3	0.267	0.038	3
FnY410A	0.221	0.010	3	1.361	0.148	3
FnFLX	0.240	0.033	3	0.334	0.051	3
FnΔLoop	0.046	0.004	3	0.062	0.007	3
LbWT	0.112	0.005	3	0.055	0.007	3
LbW355A	0.040	0.003	3	0.183	0.008	3
LbFLX	0.029	0.007	3	0.127	0.016	3
LbΔLoop	0.120	0.007	3	0.078	0.010	3
AsWT	0.084	0.017	3	0.007	0.002	3
AsW382A	0.052	0.002	3	0.035	0.010	3
AsFLX	0.076	0.025	3	0.001	3.8e-4	3
AsΔLoop	0.046	0.005	3	0.002	5.0e-5	3
AsP1153A	0.061	0.006	3	0.007	4.9e-4	3
AsFLX-2	0.034	0.005	3	0.003	0.001	3

Table S2: *Mean rates of NTS and TS cleavage of plasmid DNA target, with standard deviation, and n.*

**Cas12a**	**Amino acids**	**Mutation**
FnFLX	D1158-D1164	GSGSGSG substitution (inclusive)
FnΔLoop	N1153-T1165	Deletion (inclusive)
LbFLX	K1079-D1085	GSGSGSG substitution (inclusive)
LbΔLoop	R1073-E1088	Deletion (inclusive)
AsFLX	E1165-F1169	GSGSG substitution (inclusive)
AsΔLoop	P1162-Y1173	Deletion (inclusive)
AsP1153A	P1153	Substitution to alanine
AsFLX-2	E1144-I1155	Truncation/substitution to GSG
**AsΔLoop-2*	*D1137-A1156*	*Deletion (inclusive)* ***no protein expression**

Table S3: *Mutations made to disrupt Nuc-loops of Cas12a orthologues.*

Orthologue	**Domain**	**Residue #**
FnCas12a	REC2	405-445; 463-469
	Nuc-loop	1154-1162
	NUC	1097-1104; 1196-1203
LbCas12a	REC2	345-394
	Nuc-loop	1076-1085
	NUC	1018-1027; 1117-1127
AsCas12a	REC2	382-414
	Nuc-loop	1169-1172
	NUC	1088-1096; 1202-1215

Table S4: *Protein contacts involved in REC2-Nuc 'clamping', from MD simulation data.*

	Sequence	T°(a)
**Vector subcloning**		
fn_codingseq_f	AGATATACATATGAGCATCTACCAGGAG	61
fn_codingseq_r	TCTTCTTTGGGGCATAGTCGGGGACATC	61
as_codingseq_f	AGATATACATATGACACAGTTCGAGGGC	64
as_codingseq_r	GGGACATCATTTAGGCATAGTCGGGGAC	64
pet_fninsert_f	CGACTATGCCCCAAAGAAGAAGCGGAAG	59
pet_fninsert_r	AGATGCTCATATGTATATCTCCTTCTTAAAGTTAAAC	59
pet_asinsert_f	CTATGCCTAAATGATGTCCCCGACTATG	58
pet_asinsert_r	ACTGTGTCATATGTATATCTCCTTCTTAAAGTTAAAC	58
**Mutagenesis**		
fn_y410a_f	GTTTGATGACGCTTCCGTGATTGGGACC	66
fn_y410a_r	ACCTGCTGTGACAGGTCT	66
fn_e1006a_f	GTGGTGTTCGCGGATCTGAAC	58
fn_e1006a_r	AATGGCATTGTATTCGATG	58
fn_nucLOOPdel_f	AGGGAGGTGTACCCAACC	65
fn_nucLOOPdel_r	GATCAGGCGAGATCCGAAG	65
fn_flexstem_f	CGGGTCCGGGACTAGGGAGGTGTACCCA	62
fn_flexstem_r	GACCCGGACCCGGAATTTCGAAAGTTGATCAGG	62
as_w382a_r	AGGGCGCTGCTGATTGTC	68
as_w382a_f	GTGCGACCACGCGGATACACTGAGGAATG	68
as_e993a_f	GGTGGTGCTGGCCAACCTGAATTTC	64
as_e993a_r	ACGGCCTGGTAGTGGATC	64
as_p1153a_f	CAAGGGCACCGCGTTCATCGCCG	67
as_p1153_r	GCGTCAAACTGTGTCTCGTTC	67
as_flx2_f	TGGCGCCGGCAAGAGAATCGTG	65
as_flx2_r	GAGCCGTTCTTCTCGAACACGATATCCC	65
as_nucLOOPdel_f	CGGGACCTGTATCCTGCC	67
as_nucLOOPdel_r	CACGATTCTCTTGCCGGC	67
as_flexstem_f	CGGGTCCACCGGCAGATACCGGGAC	69
as_flexstem_r	GACCCGGAGATCACTGGCACGATTCTCTTGC	69
lb_w355a_f	CTTCGGCGAGGCGAACGTGATC	56
lb_w355a_r	ATATCCTTGGAGATTGTG	56
lb_e925a_f	ATCGCCCTGGCGGACCTGAAC	64
lb_e925a_r	CACGGCATCGTACTTCTCCAC	64
lb_nucLOOPdel_f	GTGTGCCTGACCAGCGC	69
lb_nucLOOPdel_r	GATCCGGTTGCCGTAGGAG	69
lb_flxstem_f	CGGGTCCGGGTGGGAGGAGGTGTGCCTG	68
lb_flxstem_r	GACCCGGACCCAGGATTCCGGAAGATTCTGATCCGG	68

Table S5: Sequences of cloning primers.

Name	Sequence
Mini_locus_top	ATAAGGAGATATACCATGGGAATTTCTACTGTTGTAGATTATGGGTATAAATGGGCTCGCGAAATTTCTACTGTTGTAGATTATGGGTATAAATGGGCTCGCGAAATTTCTACTGTTGTAGATGAATTCGAGCTCGGCGCGCC
Mini_locus_bottom	GGCGCGCCGAGCTCGAATTCATCTACAACAGTAGAAATTTCGCGAGCCCATTTATACCCATAATCTACAACAGTAGAAATTTCGCGAGCCCATTTATACCCATAATCTACAACAGTAGAAATTCCCATGGTATATCTCCTTAT
FQ ssDNA	5'-/56-FAM/TTT TTT TTT/ZEN/TTT/3IaBkFQ/ -3'
TS	CCCGGTGTCACGCCACTTGACAGGCGAGTAACAGACATGGACCATCAGGAAACATTAACGTACTGATGTTAACAGCTGACCCAATAAGTGGCAGAG
TS_truncated	TAACAGACATGGACCATCAGGAAACATTAACGTACTGATGTTAACAGCTGACCCAATAAGTGGCAGAG

Table S6: Oligonucleotide sequences used in 'locus' cloning and *trans* cleavage assays.

**crRNAs for in vitro cis and trans cleavage assays**	
Lb_23	UAAUUUCUACUAAGUGUAGAUCUGAUGGUCCAUGUCUGUUACUC
Lb_20	UAAUUUCUACUAAGUGUAGAUCUGAUGGUCCAUGUCUGUUA
As_23	UAAUUUCUACUCUUGUAGAUCUGAUGGUCCAUGUCUGUUACUC
As_20	UAAUUUCUACUCUUGUAGAUCUGAUGGUCCAUGUCUGUUA
Fn_23	UAAUUUCUACUGUUGUAGAUCUGAUGGUCCAUGUCUGUUACUC
Fn_20	UAAUUUCUACUGUUGUAGAUCUGAUGGUCCAUGUCUGUUA
**crRNAs for human cell gene editing via RNP electroporation**	
Lb_DNMT1-3	UAAUUUCUACUAAGUGUAGAUCUGAUGGUCCAUGUCUGUUACUCG
Lb_DNMT1-7	UAAUUUCUACUAAGUGUAGAUGCUCAGCAGGCACCUGCCUCAGCU
Lb_AGBL1	UAAUUUCUACUAAGUGUAGAUGAUUGAAGGAAAAGUUACAAAGGU
As_DNMT1-3	UAAUUUCUACUCUUGUAGAUCUGAUGGUCCAUGUCUGUUACUCG
As_DNMT1-7	UAAUUUCUACUCUUGUAGAUGCUCAGCAGGCACCUGCCUCAGCU
As_AGBL1	UAAUUUCUACUCUUGUAGAUGAUUGAAGGAAAAGUUACAAAGGU
Fn_DNMT1-3	UAAUUUCUACUGUUGUAGAUCUGAUGGUCCAUGUCUGUUACUCG
Fn_DNMT1-7	UAAUUUCUACUGUUGUAGAUGCUCAGCAGGCACCUGCCUCAGCU
Fn_AGBL1	UAAUUUCUACUGUUGUAGAUGAUUGAAGGAAAAGUUACAAAGGU

Table S7: RNA oligonucleotide sequences.

Site name	site sequence	primer set name*	Forward primer	Reverse primer
dnmt1-3	TTTCCTGATGGTCCATGTCTGTTA	DNMT1-3 / TTTC-2	TCCCTTAGCACTCTGCCACTTAT	GTTAAAAACACAACATCAGTGCATGT
dnmt1-7	TTTGGCTCAGCAGGCACCTGCCTC	DNMT1-7 /TTTG-2	AGCAGGCCTTTGGTCAGGTT	AGACATGGACCATCAGGAAACAT
agbl1	TTTAGATTGAAGGAAAAGTTACAA	AGBL1 /TTTA-6	GAAGAGAAATCTGCGTGGAGAGA	GAAAACCCCCAAAAATCCCA
dnmt1-3-OT1	TTTCCTGATGGTCCATGTCTGAAT	WT-AsCas12a-TTTC-2-chr1	CCTATTCTTCCGCCATTTTCC	CTGAATGCCTGCTATGTACACACA
dnmt1-3-OT2	TTTCCTGATGGTCCACATCTGTTA	enAsCas12a-TTTC-2-chr11	TGCCACATTACCCTCTAAAAGTCA	TGCGCAGTGTGTTTAGGAAGTT
dnmt1-3-OT3	TTTCCTGATGGTCCATATCTGTGG	enAsCas12a-TTTC-2-chr10	TCAGGTGATGGTTTGGCAATC	ATAGAAAGCCTCCCCACCTAAGG
dnmt1-3-OT4	TTTCCTGATGGTCCATACCTGTTA	enAsCas12a-TTTC-2-chrX	CTCTTCCCCTCAACCACTAAATATG	TCTGAGGTTTTCTTCCCTTTTCC
dnmt1-7-OT1	AGCAGCTCAGCAGGCACCTGCCTT	enAsCas12a-TTTG-2-chr21	CAGCCAGGGCCTCATTAAAC	CATGTTGAATGTTGGCACGAA
dnmt1-7-OT2	AACAGCTCAGCAGACACCTGCCAA	enAsCas12a-TTTG-2-chr2	TTGCCATGACAACAACTCAGG	GGACGCAATGTCAGCTGTGT
dnmt1-7-	TTTAGCTCAGCTGACACCTGCCCA	enAsCas12a-TTTG-	TGTTGCATAGGCTTGTTCTGTTC	TTGTTAGGGTTCATTGTAGCTGACA

OT 3		2-chr12		
abg11-OT 1	TTTAGATTAATGGAAAAGTTACAA	enAsCas12a-TTTA-6-chr6	CACTGCACCCGGCCATA	TGTGGAGTCTTGAACTGGATCTTG
abg11-OT 2	TTTAGACTTAAAGAAAAGTTACAA	enAsCas12a-TTTA-6-chr8	GTGGTAGTACTTCCTTCATACTGAGCA	GTCCCGTGGAGTAGTTGATGTG
abg11-OT 3	CTTGGATTAAAGGAAAAGCTACAA	enAsCas12a-TTTA-6-chr10	TGGAAATAAGCAATCAGGCCTT	TTTGTTTTGTCTTTCCTTACATCGTT

Table S8: Primers for high-throughput sequencing. *Sites names as per Kleinstiver 2019

"Kleinstiver, B.P., Sousa, A.A., Walton, R.T. et al. Engineered CRISPR–Cas12a variants with increased activities and improved targeting ranges for gene, epigenetic and base editing. Nat Biotechnol 37, 276–282 (2019). https://doi.org/10.1038/s41587-018-0011-0"
